# The Fanconi Anemia Pathway Inhibits mTOR Signaling and Prevents Accelerated Translation in Head and Neck Cancer Cells

**DOI:** 10.3390/cancers17152583

**Published:** 2025-08-06

**Authors:** Bianca Ruffolo, Sara Vicente-Muñoz, Khyati Y. Mehta, Cosette M. Rivera-Cruz, Xueheng Zhao, Lindsey Romick, Kenneth D. R. Setchell, Adam Lane, Susanne I. Wells

**Affiliations:** 1 Division of Oncology, Cincinnati Children’s Hospital Medical Center, Cincinnati, OH 45229, USA; bianca.ruffolo@cchmc.org (B.R.); cosette.riveracruz@cchmc.org (C.M.R.-C.); 2 Department of Pediatrics, College of Medicine, University of Cincinnati, Cincinnati, OH 45229, USA; xueheng.zhao@cchmc.org; 3 Division of Pathology and Laboratory Medicine, Cincinnati Children’s Hospital Medical Center, Cincinnati, OH 45229, USA; sara.vicentemunoz@cchmc.org (S.V.-M.); lindsey.romick-rosendale@cchmc.org (L.R.); kenneth.setchell@cchmc.org (K.D.R.S.); 4 Department of Pathology and Laboratory Medicine, College of Medicine, University of Cincinnati, Cincinnati, OH 45229, USA; 5 Division of Biomedical Informatics, Cincinnati Children’s Hospital Medical Center, Cincinnati, OH 45229, USA; 6 Department of Biomedical Informatics, College of Medicine, University of Cincinnati, Cincinnati, OH 45229, USA; 7 Division of Bone Marrow Transplantation and Immune Deficiency, Cincinnati Children’s Hospital Medical Center, Cincinnati, OH 45229, USA; adam.lane@cchmc.org

**Keywords:** cancer metabolism, Fanconi Anemia, head and neck cancer, mTOR

## Abstract

**Background/Objectives:** The Fanconi anemia (FA) pathway is essential for the repair of DNA interstrand crosslinks and maintenance of genomic stability. Germline loss of FA pathway function in the inherited Fanconi anemia syndrome leads to increased DNA damage and a range of clinical phenotypes, including a heightened risk of head and neck squamous cell carcinoma (HNSCC). Non-synonymous FA gene mutations are also observed in up to 20% of sporadic HNSCCs. The mechanistic target of rapamycin (mTOR) is known to stimulate cell growth, anabolic metabolism including protein synthesis, and survival following genotoxic stress. **Methods/Results:** Here, we demonstrate that FA− deficient (FA−) HNSCC cells exhibit elevated intracellular amino acid levels, increased total protein content, and an increase in protein synthesis indicative of enhanced translation. These changes are accompanied by hyperactivation of the mTOR effectors translation initiation factor 4E Binding Protein 1 (4E-BP1) and ribosomal protein S6. Treatment with the mTOR inhibitor rapamycin reduced the phosphorylation of these targets and blocked translation specifically in FA− cells but not in their isogenic FA− proficient (FA+) counterparts. Rapamycin-mediated mTOR inhibition sensitized FA− but not FA+ cells to rapamycin under nutrient stress, supporting a therapeutic metabolism-based vulnerability in FA− cancer cells. **Conclusions:** These findings uncover a novel role for the FA pathway in suppressing mTOR signaling and identify mTOR inhibition as a potential strategy for targeting FA− HNSCCs.

## Simple Summary

The Fanconi anemia (FA) pathway is essential for DNA repair and maintenance of genome integrity. Individuals born without a functional FA pathway are at high risk of developing head and neck cancer early in life, often with poor prognosis. We sought to define whether a nonfunctional FA pathway affects cellular metabolism and related signal transduction. FA loss stimulated amino acid accumulation, translation, and protein production via mTOR signaling, which is reported to promote cancer development and progression. Pharmacological mTOR inhibition using rapamycin suppressed these pro-cancer phenotypes, suggesting that targeting protein synthesis may offer a therapeutic avenue for the management of FA− associated cancers.

## 1. Introduction

The Fanconi anemia (FA) pathway provides a crucial DNA repair mechanism responsible for recognizing and resolving DNA interstrand crosslinks (ICLs) [1]. This pathway operates through three complexes comprising over 20 interacting proteins [2]. The core complex, once assembled with its eight constituent proteins, functions as an E3 ubiquitin ligase, monoubiquitinating the FA intermediate (ID2) complex, which consists of FANCI and FANCD2 [3,4,5]. This monoubiquitination is essential for the functionality of the FA pathway. Subsequently, a downstream complex utilizes the homologous recombination (HR) repair machinery for appropriate DNA repair [6,7,8]. The loss-of-function mutation of any component of this pathway results in a failure of ID2 complex monoubiquitination and/or HR repair failure [1,9,10]. Consequently, loss of the FA pathway can lead to genomic instability with a pronounced accumulation of structural variants [11,12].

The significance of the FA pathway becomes particularly evident when examining individuals with loss-of-function germline mutations in FA genes [1]. Those born with FA pathway defects develop the disease FA, a condition marked by various clinical manifestations, including developmental abnormalities and bone marrow failure [13]. Notably, amongst other phenotypes, these individuals face a dramatically elevated risk of developing squamous cell carcinomas (SCCs) in the head and neck, anogenital tract, and skin [14,15,16]. In the case of head and neck SCC (HNSCC), patients with FA often experience particularly aggressive disease progression, characterized by multifocal, recurrent, and high-grade tumors [12,17,18,19]. Of note, approximately 20% of individuals in the general population who develop HNSCCs harbor at least one non-synonymous mutation in the FA pathway, often in ID2 and HR components, highlighting a potential role in sporadic cancer development and/or progression [20,21,22,23].

Given the crucial role of the FA pathway in SCC prevention, it is important to understand cellular processes regulated by this pathway. Previously published data suggest that FANCD2 and FANCJ knockdown in HNSCC cell lines enhances aggressive cancer phenotypes, such as epithelial-to-mesenchymal (EMT)-like morphological changes and increased invasion, at least in part due to metabolic dysregulation [24,25,26]. Herein, we report that FA− deficient versus -proficient cells harbor increased amino acid and protein content associated with elevated translation. Protein upregulation has previously been linked to aggressive cancer phenotypes in other model systems, notably by increasing the ability of cancer cells to rapidly generate the macromolecules required for unregulated growth, invasion, and metastasis [27,28,29].

Among the key regulators of amino acid homeostasis and protein synthesis in cancer is the mechanistic target of rapamycin (mTOR) signaling pathway. This pathway is comprised of two distinct complexes, mTORC1 and mTORC2, which regulate vital processes including cellular metabolism and growth [30]. mTORC1 functions as a nutrient sensor, modulating anabolic and catabolic activities based on cellular requirements, while mTORC2 predominantly governs cellular growth and proliferation [30]. We show that FA deficiency leads to increased phosphorylation of mTORC1 effectors, including ribosomal protein S6 and 4E Binding Protein 1 (4E-BP1), which are known to enhance translational output [31,32]. Importantly, pharmacological inhibition of mTORC1 with rapamycin in FA− deficient HNSCC cells reduced phosphorylation of these targets, suppressed translation, and impaired cellular growth under nutrient stress. These findings provide new insights into the tumor-suppressive functions of the FA pathway and identify mTOR signaling as a therapeutic vulnerability in FA− deficient cancers.

## 2. Materials and Methods

### 2.1. Cell Lines and Culture Conditions

Human HPV-negative HNSCC cell lines (UMSCC1 and UMSCC22b), HNSCC cell lines derived from patients with FA (VU-1365 and OHSU-974), and normal immortalized keratinocytes derived from foreskin (NIKS) and mucosa (NOKS) were maintained as previously described [33,34,35]. NIKS were cultured with irradiated J2-3T3 mouse fibroblast feeder cells. Knockdown of FANCD2 (D2sh) was performed with a lentiviral (short-hairpin) shRNA and a non-targeting (NTsh) control RNA, as previously described [34,36]. Complementation of FANCA was performed via the S91 lentiviral vector, as previously described [34]. Transduced cells were maintained in 1.25 μg/mL of puromycin (shRNA treated) or 0.8 mg/mL of geneticin (S91 lentiviral vector treated), and cell lines were subjected to routine confirmation of FANCD2 knockdown and FANCA correction.

### 2.2. Metabolomic Analysis

#### 2.2.1. Cell Collection and Metabolite Extraction

Upon reaching confluency, the culture media was removed, and the cells were rapidly washed three times with ice-cold phosphate-buffered saline (PBS). Metabolic activity was quenched with cold acetonitrile (CH_3_CN), with the subsequent addition of nano-pure water (CH_3_CN/H_2_O at 2:1.5 (*v*/*v*)) to enable efficient cell harvesting. Intracellular metabolites were extracted using CH_3_CN/H_2_O/CHCl_3_ (2:1.5:1 (*v/v/v*)) [37]. Following phase separation, polar metabolites were concentrated through lyophilization (CentriVap Labconco, Kansas City, MO, USA) while non-polar compounds were evaporated in a SpeedVac device. The pellet residues from the extraction were washed with pure methanol (500 µL) and centrifuged (12000× *g* at 4 °C for 10 min). After discarding the resulting supernatant, the remaining pellets were dried in a SpeedVac and subsequently processed for DNA extraction for normalization purposes.

#### 2.2.2. Nuclear Magnetic Resonance (NMR) Spectroscopy and Data Analysis

NMR experiments were performed at 288 K using a Bruker Avance III HD 600 MHz spectrometer (Bruker Biospin, Billerica, MA, USA) equipped with a 5 mm Broadband Observed (BBO) Prodigy probe. Polar metabolites from cells were reconstituted in 220 μL of NMR buffer (100 mM phosphate buffer, pH 7.3, containing 1 mM trimethylsilyl propionic acid-d4 sodium salt (TSP) as an internal standard and 1 mg/mL of sodium azide in 100% deuterium oxide (D_2_O)). Samples (200 μL) were transferred to a 3 mm NMR tube for analysis. One-dimensional (1D) ^1^H spectra were acquired using the noesygppr1d pulse sequence with water signal suppression (25 Hz bandwidth presaturation). ^1^H spectra were collected with 512 transients using a spectral width of 15 ppm, with a relaxation delay of 4.0 s and an acquisition time of 2.0 s. The ^1^H spectra were then linear predicted and zero filled to 128 k points and apodized with 1 Hz exponential line broadening.

All NMR data were acquired and transformed using TOPSPIN 3.5 (Bruker BioSpin), then processed (phased and baseline corrected) and analyzed with MestReNova software (MNova 12.0.3, Santiago de Compostela, Spain). ^1^H chemical shifts were internally referenced to the methyl resonance of the TSP peak at 0.00 ppm. Peak integration was performed using the global spectra deconvolution (GSD) algorithm in MNova software. Metabolite quantification was achieved by correcting integrated peak areas for the number of contributing protons and calibrated against the TSP internal standard. Finally, the nmoles of metabolites were normalized to DNA content from the extraction residue pellets to account for variation in the biological material.

Metabolites were identified through comparison with an in-house database, public repositories including HMDB (Human Metabolome database [38]) and BMRB (Biological Magnetic Resonance Data Bank [39]), and literature reports [40,41]. For selected samples, two-dimensional (2D) ^1^H-^1^H TOtal Correlation SpectroscopY (TOCSY) and 2D ^1^H-(^13^C) Heteronuclear Single Quantum Correlation (HSQC) experiments were recorded to confirm metabolite assignments.

#### 2.2.3. Mass Spectrometry (MS) and Data Analysis

Untargeted metabolomics profiling was conducted on a Q ExactiveTM plus hybrid quadrupole-OrbitrapTM mass spectrometer interfaced with a Vanquish Ultra-High Performance Liquid Chromatography (UHPLC) system (Thermo Scientific, Waltham, MA, USA). A gradient mobile phase was used with a binary solvent system, which changed from 85% solvent B (acetonitrile), was held for 2 min, then to 80% B at 3 min, 80% B at 5 min, 75% B at 6 min, 75% B at 7 min, 70% B at 8 min, 70% B at 9 min, 50% B at 10 min, 50% B at 12 min, 25% B at 13 min, 25% B at 16 min, then to 100% solvent A (20 mM ammonium acetate + 20 mM ammonium hydroxide in 95:5 water/acetonitrile, pH 9.45) at 18 min, then held for 5 min, changed to 85% solvent B over 1 min, and then this was held for 6 min. The total run time was 30 min, and the flow rate was 0.15 mL/min. Solvent A consisted of 95:5 water/acetonitrile, with 20 mM ammonium acetate + 20 mM ammonium hydroxide, pH 9.45; solvent B consisted of acetonitrile. An Xbridge BEH Amide (150 × 2.1 mm, 2.5 u, 130 A pore size) HPLC column (Waters, Milford, MA, USA) was used.

The electrospray ionization (ESI) source was operated in the following parameters: spray voltage, 2.5 KV; capillary temperature, 350 °C; sheath gas flow rate, 35; auxiliary gas heater temperature, 325 °C. Data were acquired using a full MS scan (mass scan range 73–1000 mass-to-charge (*m*/*z*)), AGC target 3e6, maximum IT 100 ms, resolution 140,000) and collision-induced dissociation-based data dependent on MS/MS (resolution 17,500, AGC target 1 × 10^5^, maximum IT 50 ms, loop count 15, top N = 15, isolation window 1.0 *m*/*z*, stepped NCE 25, 35, 45).

UHPLC-HRMS raw data (.RAW) from both electrospray positive and negative ionization modes were converted to .mzML files using ProteoWizard’s msConvert [42] tool. Subsequent files were uploaded into El-MAVEN [43,44,45] for data deconvolution and interpretation. Each ion feature, defined as a deconvoluted peak in the mass chromatogram, was annotated by its retention time and mass-to-charge ratio (*m*/*z*). Metabolite identities were assigned by matching accurate *m*/*z* values and fragmentation patterns to entries in the Human Metabolome Database (HMDB). 

### 2.3. Translation and Proliferation Assays

Cells were plated on poly-L-lysine-coated coverslips in basal media and grown to 70–80% confluency. Prior to reagent addition, cells were cultured for 30 min in methionine-free media. Subsequently, the media were replaced with 50 µM L-homopropargylglycine (HPG) or 10 μM 5-ethynyl-2′-deoxyuridine (EdU) in methionine-free media, and the cells were incubated for 3 h. In relevant experiments, 3 µM rapamycin, or an equivalent volume of DMSO control, was added to both HPG- and EdU-containing groups after 30 min of the initial methionine-free media incubation. After 3 h, cells were fixed in 4% paraformaldehyde (PFA) for 20 min and rinsed with PBS. The cells were then stored in PBS at 4 °C for subsequent immunofluorescent staining.

### 2.4. Immunofluorescence (IF)

Coverslips were permeabilized for 3 min at room temperature with 0.2% Triton X-100. The reaction mixture for the Click-iT EdU Cell Proliferation Kit for Imaging, Alexa Fluor 647 dye (Thermo Fisher Scientific C10340, Waltham, MA, USA), and the Click-iT HPG Alexa Fluor 488 Protein Synthesis Assay Kit (Thermo Fisher Scientific C10428) were prepared as per the manufacturer instructions. Coverslips treated with EdU were blocked with 10% normal donkey serum (Jackson ImmunoResearch 017-000-121, West Grove, PA, USA) dissolved in antibody dilution buffer (3% BSA, 0.3% Triton X-100, 0.05% Tween 20, 0.04% sodium azide) for 1 h at room temperature. A primary antibody solution was made with 0.25 µM cleaved caspase-3 Asp175 primary antibody (Cell Signaling 9661, Beverly, MA, USA) and 2% normal donkey serum in antibody dilution buffer, and the coverslips were incubated with this mixture for 1 h at 37 °C. A secondary antibody dilution was made with 4 µg/mL of AffiniPure Alexa Fluor^®^ 488 Anti-Rabbit IgG (H + L) Donkey Secondary Antibody (Jackson ImmunoResearch 711-545-152) and a 1:1000 dilution of Wheat Germ Agglutinin (WGA) Alexa Fluor 568 Conjugate (Invitrogen W56133, Waltham, MA, USA). For coverslips treated with both HPG and EdU, the secondary mixture contained a 1:5000 dilution of Hoechst 33,342 (Thermo Fisher Scientific C10340) in antibody dilution buffer, and the coverslips were fixed for confocal imaging with ProLong Gold Antifade Mountant (Thermo Fisher Scientific P36930).

### 2.5. BCA and Western Blot Analysis

A total of 1 million cells were plated in 10 cm dishes and cultured for 2 days until confluency reached 80–90%. Cells were then treated with 3 µM rapamycin or equivalent DMSO for 24 h. Cells cultured in basal media were used as an untreated control. Untreated, DMSO-treated, or rapamycin-treated cells were harvested in PBS using a cell scraper. For total protein assays, cells were first counted and separated into 2 million cell pellets. All resulting cell pellets were re-suspended in RIPA buffer (10% SDS, 1 M Tris, 5 M NaCl, 0.25 M EDTA, 1% Triton X-100, 0.5% sodium deoxycholate) with HALT protease, phosphate inhibitor cocktail (Thermo Fisher Scientific 78443) and 200 µM Na_3_O_4_V. Pellets were lysed with 30 amplitude sonication for 10 s on a Fisherbrand Model CL-18 sonicator. The protein concentration of the lysates was determined using Pierce™ BCA Protein Assay Kits (Thermo Fischer Scientific 23225). For Western blot analysis, 40 μg of protein was loaded per lane on 4–20% Bis-Tris gels (Bio-Rad 5678094, Hercules, CA, USA), and the protein was transferred to a Transblot turbo midi-size membrane (Bio-Rad 1704275) at 80 V for 2 h. Membranes were blocked in 5% bovine serum albumin (BSA) in Tris-buffered saline with Tween 20 (TBST). Primary antibodies were dissolved in 5% BSA with 0.3% sodium azide at a 1:1000 dilution. Primary antibodies included the following: eIF2α (Cell Signaling 9722), phospho-eIF2α Ser51 (Cell Signaling 9721), eIF4 (Cell Signaling 9742), phospho-eIF4 Ser209 (Cell Signaling 9741), 4E-BP1 (Cell Signaling 9452), phospho-4E-BP1 Thr37/46 (Cell Signaling 2855), S6 ribosomal protein (Cell Signaling 2317), and phospho-S6 ribosomal protein Ser235/236 (Cell Signaling 4858). Membranes were washed in TBST and incubated with ECL anti-rabbit (Thermo Fisher Scientific NA934V) or ECL anti-mouse (Thermo Fisher Scientific NA931V) secondary antibodies for 1 h at room temperature. Secondary antibody solution was made in 5% BSA with Rhodamine Anti-actin antibody (Bio-Rad 12004163) at a dilution of 1:5000 as a loading control. Blots were imaged on a Bio-Rad ChemiDoc Imager with Western Lightening ECL reagent (PerkinElmer NEL103001E, Shelton, CT, USA).

### 2.6. CellTiter-Fluor Viability Assay

Cells were plated on 96-well plates and seeded at 10,000 cells/well in technical replicates of six per sample with media-only controls. Rapamycin (or the DMSO equivalent) was added 24 h after plating in either High Glucose Gibco DMEM (Thermo Fisher Scientific 11-965-118) with serum or Gibco MEMα without serum (Thermo Scientific 12-571-063) for 6, 24, 48, or 72 h. The CellTiter-Fluor Cell Viability Assay was performed three times separately as per the manufacturer protocol (Promega G6082, Madison, WI, USA).

### 2.7. Statstical Analysis

Statistical analyses were conducted using GraphPad Prism 9 software (GraphPad Software, San Diego, CA, USA), with an alpha value set at 0.05. Technical replicates were averaged to represent the value for each biological replicate data point. Results are presented as the mean of at least three technical replicates, with standard deviation indicating variation within the dataset. All data passed the Shapiro–Wilk test for normality (*p* > 0.05). Comparisons between NTsh and D2sh were performed using either Student’s *t*-test or Welch’s *t*-test, depending on the outcome of Leven’s test for homogeneity of variances. Studies involving rapamycin treatment were analyzed using a two-way analysis of variance (ANOVA), followed by Sidak’s multiple comparisons test. Dose–response and growth curves were analyzed using two-way ANOVA tests for each rapamycin concentration, with significant differences determined by an F-test.

## 3. Results

### 3.1. FA Pathway Loss in HNSCC Cells Increases Total Protein and Amino Acid Levels

**Figure 1:**
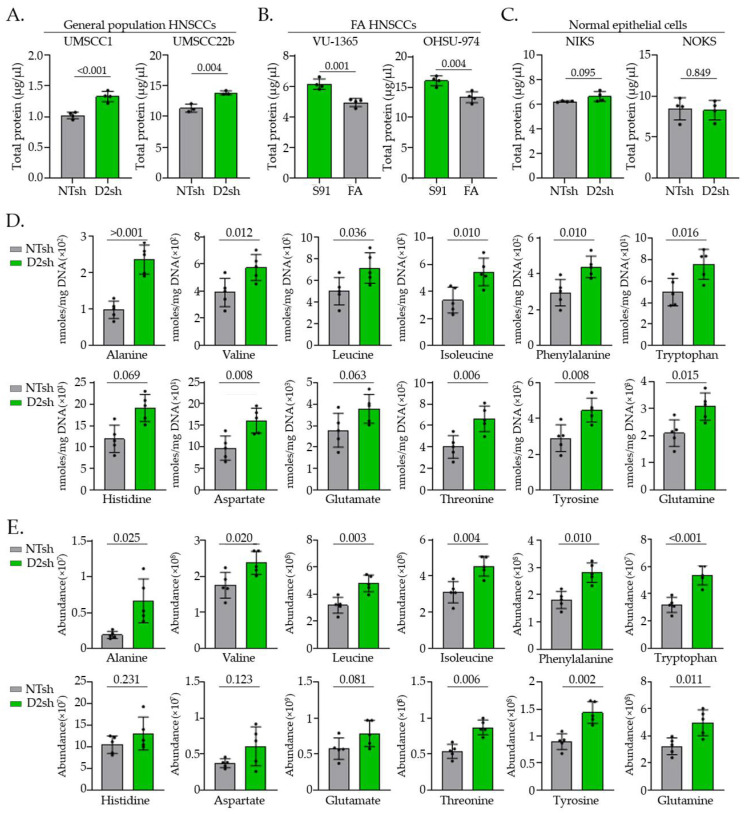
FA pathway loss increases protein and amino acid levels in HNSCC cells. (**A**–**C**) Total protein levels were measured using the Bicinchoninic Acid (BCA) assay in 2 million cells across a panel of cell lines. (**A**,**C**) Sporadic HNSCC and normal immortal keratinocytes were subjected to FANCD2 knockdown (D2sh) and compared to FA− proficient controls (NTsh). (**B**) HNSCCs derived from patients with FA were complemented with the missing FANCA gene (FA) and compared to the FA− deficient empty vector control (S91). (**D**) NMR quantification of amino acids in NTsh (FA− proficient) and D2sh (FA− deficient) UMSCC1 cells. (**E**) MS quantification of amino acids in NTsh and D2sh UMSCC1 cells showing the absolute abundance of ions corresponding to each detected peak. Metabolomics data are presented as mean ± SD (*n* = 5), with statistical significance determined using Welch’s *t*-test. Total protein data are presented as mean ± SD (*n* = 4), with statistical significance determined using Student’s *t*-test.

During sample preparation for protein detection, we consistently observed higher overall protein concentrations in FA− deficient (FA−) samples than in their FA− proficient (FA+) counterparts despite equal cell numbers (Figure 1A,C). We tested whether the increase in total protein was consistent across multiple FA− isogenic cell lines and systems. Sporadic HNSCC cells exhibited elevated total protein levels upon FANCD2 knockdown (Figure 1A). Conversely, HNSCC cells from individuals with FA with an inherited FANCA gene defect responded to FANCA complementation by reducing total protein levels (Figure 1B). Importantly, normal immortalized foreskin keratinocytes (NIKS) and normal oral keratinocytes (NOKS) did not harbor increased total protein levels upon FANCD2 knockdown, indicating that the observed increase is a cancer-specific response (Figure 1C).

This prompted the targeted detection of amino acids in FA− versus FA+ cells, measured by NMR (Figure 1D) and MS (Figure 1E), using DNA content for normalization to ensure equivalent cell numbers. We observed increased levels of most amino acids using both technologies (Figure 1D,E). Together, these observations suggest that FA pathway loss in HNSCC cells stimulates protein accumulation in correlation with increased amino acid levels and that this is reversible by FA gene complementation in cancer cells derived from patients with FA.

### 3.2. FA Pathway Loss Increases Translation and Activates mTOR Signaling in HNSCC Cells

**Figure 2:**
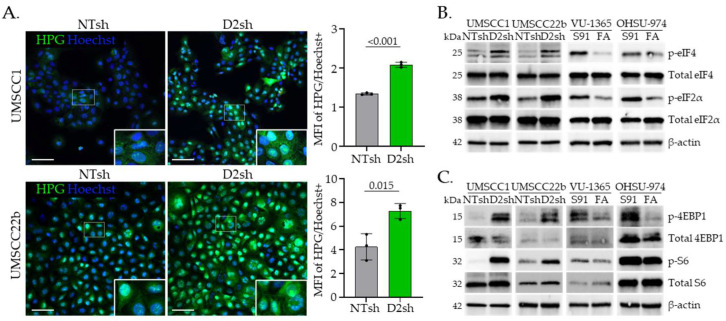
FA pathway loss increases translation and activates mTOR signaling. (**A**) Immunofluorescence (IF) images and quantification of mean fluorescence intensity (MFI) normalized to cell number (Hoechst+ cells) following 3 h incorporation of L-Homopropargylglycine (HPG) in NTsh and D2sh UMSCC1 and UMSCC22b cells. Scale bar = 100 µm. (**B**) Western blot analysis of translation initiation markers and (**C**) mTOR activation markers across the HNSCC cell lines shown in Figure 1A,B. Data are presented as mean ± SD (*n* = 3), with statistical significance determined using Student’s *t*-test.

To test whether the increased total protein content observed in FA− HNSCC cells might be due to increased translation, we used L-homopropargylglycine (HPG) to quantify protein synthesis over a 3 h period. FA− HNSCC cells exhibited a two-fold increase in protein translation compared to FA+ HNSCC cells (Figure 2A). This was accompanied by elevated phosphorylation and subsequent activation of two translation initiation factors, eIF4 and eIF2, supporting heightened translation activity in FA− HNSCC cells relative to their FA+ counterparts [46,47,48] regardless of whether FA pathway loss was sporadic or at the germline level (Figure 2B and Figure S1). To identify potential effectors of this phenotype, we examined the mTOR pathway, a well-established driver of translation in cancer [49], which is known to phosphorylate 4E-BP1 and ribosomal protein S6 to enhance translation efficiency [50]. Increased activation of these proteins, as well as eIF4 and eIF2, following FA pathway loss was detected in both sporadic and germline FA− tumors (Figure 2C and Figure S2). These findings suggest that FA pathway loss in HNSCCs activates mTOR to stimulate translation.

### 3.3. Rapamycin Suppresses Protein Translation in FA− Deficient HNSCC Cells

**Figure 3:**
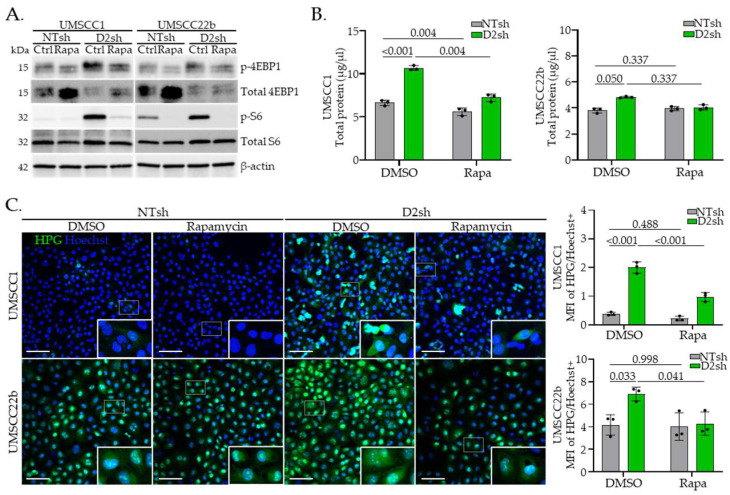
Rapamycin suppresses translation in FA− deficient HNSCC cells. (**A**) Western blot analysis of mTOR markers following 24 h treatment with 3 μM rapamycin or DMSO control. (**B**) Quantification of total protein content using BCA assay in 2 million cells treated for 24 h with rapamycin or DMSO control. (**C**) IF images and MFI quantification normalized to cell number (Hoechst+ cells) after 3 h HPG incorporation in NTsh vs. D2sh UMSCC1 or UMSCC22b cells treated with 3 μM rapamycin or DMSO at equivalent cell number. Scale bar = 100 µm. Data are presented as mean ± SD (*n* = 3). Statistical significance was determined using two-way ANOVA, followed by Sidak’s multiple comparisons test.

**Figure 4:**
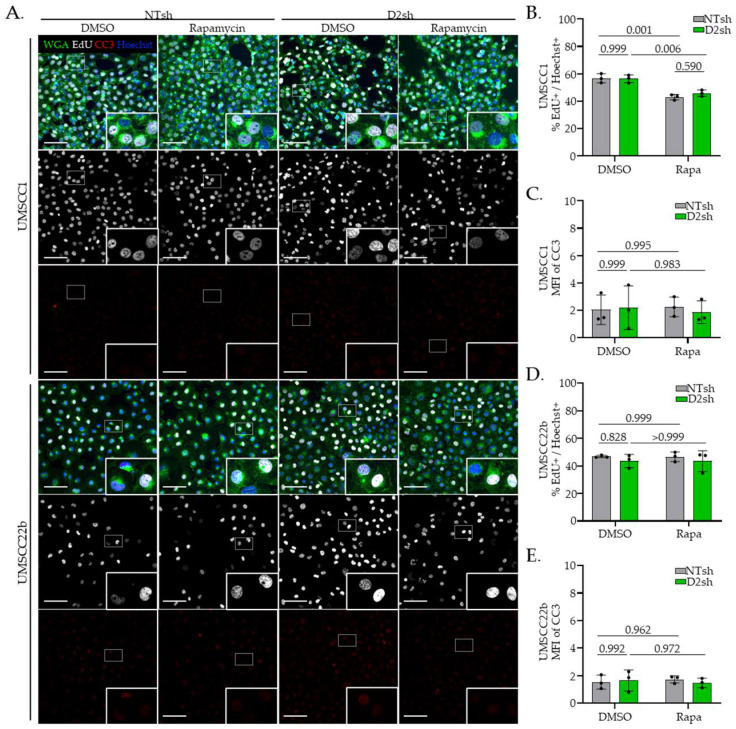
Rapamycin does not impact proliferation or apoptosis. (**A**) IF images following 3 h HPG incorporation in NTsh and D2sh UMSCC1 or UMSCC22b cells treated with 3 μM rapamycin or DMSO at equivalent cell number. (**B**,**D**) Quantification of EdU+ cells as percentage of cell number (Hoechst+ cells) per frame in NTsh and D2sh UMSCC1 (**B**) and UMSCC22b (**D**) cells. (**C**,**E**) MFI of cleaved caspase-3 (CC3) in NTsh and D2sh UMSCC1 (**C**) and UMSCC22b (**E**) cells. Scale bar = 100 µm. Data are presented as mean ± SD (*n* = 3). Statistical significance was assessed using two-way ANOVA, followed by Sidak’s multiple comparisons test.

We next tested whether the elevated translation observed in FA− cells requires mTOR signaling. The well-characterized mTOR kinase inhibitor rapamycin, which selectively blocks mTORC1 activity [51], was applied to both FA− and FA+ cells, followed by assessment of downstream mTOR signaling and total protein synthesis. After 3 h of treatment, rapamycin effectively reduced phosphorylation of 4E-BP1 and S6 in both FA− and FA+ HNSCC cells, confirming suppression of mTOR signaling. Notably, this reduction was more pronounced in FA− than in FA+ cells (Figure 3A and Figure S3). To quantify the potential effects on total protein accumulation, we treated cells with rapamycin for 24 h and observed a greater decrease in total protein levels in FA− cells than in FA+ cells (Figure 3B). Finally, HPG incorporation was significantly reduced in FA− versus FA+ cells following 3 h of rapamycin treatment (Figure 3C). Importantly, translational suppression occurred in the absence of effects on proliferation or cell death within the 3 h assay window (Figure 4A–E). Together, these data indicate that heightened translation and protein accumulation in FA− HNSCC cells is driven by increased mTOR signaling.

### 3.4. Rapamycin Inhibits FA− HNS Cell Growth Under Nutrient-Depleted Conditions

**Figure 5:**
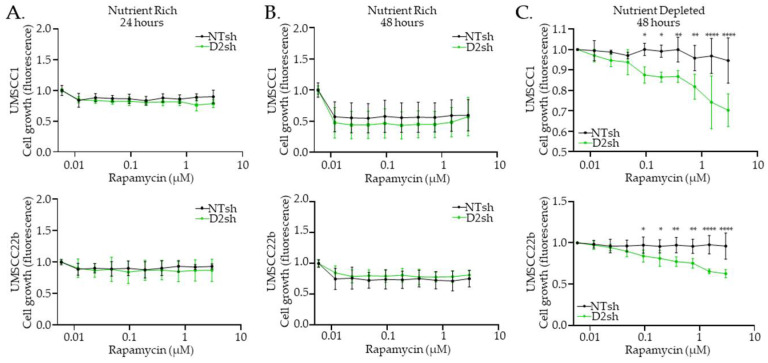
Rapamycin inhibits FA− HNSCC cell growth under nutrient-depleted conditions. Cell viability measured by CellTiter-Fluor assay after (**A**) 24 h and (**B**) 48 h of rapamycin or DMSO treatment in nutrient-rich media (DMEM) and (**C**) after 48 h in nutrient-depleted media (MEMα). Data are shown as mean ± SD (*n* = 3). Differences in growth curves were assessed using two-way ANOVA, comparing NTsh and D2sh growth at each concentration of rapamycin. Statistical significance: *p*-value <0.05 (*), <0.01 (**), and <0.0001 (****). Absence of a symbol indicates no significant difference.

We next quantified the phenotypic consequences of rapamycin in FA− versus FA+ HNSCC cells that were either cultured in nutrient-rich or nutrient-depleted media. The FA status did not affect cellular growth in response to rapamycin treatment at 6, 24, 48, or 72 h under nutrient-rich conditions (Figure 5A,B and Figure S4A–C). Cells were then treated with rapamycin in a nutrient-depleted medium, thereby simulating the amino acid- and growth factor-limited conditions characteristic of the tumor microenvironment. Under these conditions for 48 h, FA− HNSCC cells exhibited sensitivity to rapamycin treatment, while their FA+ counterparts did not (Figure 5C). This suggests that, under conditions of low nutrient availability, FA− cancer cells become reliant on mTOR-mediated translation. Rapamycin targets this vulnerability, thereby inhibiting growth. This dependency highlights a potential therapeutic window for rapamycin in treating FA− HNSCCs.

## 4. Discussion

Our findings reveal a novel role for the FA pathway in the control of mTOR signaling in HNSCCs. Specifically, we demonstrated that FA pathway loss stimulates amino acid accumulation, mTOR signaling, protein translation, and total protein concentration. Blocking mTOR activation with rapamycin reduced protein translation and total protein levels and suppressed the growth of FA− deficient HNSCC cells under nutrient-deprived conditions. This suggests that, beyond its established role in genome stabilization, the FA pathway controls protein production, potentially linking a classical genomic maintenance pathway with control of biomass production.

Traditionally, the FA pathway is recognized for its role in DNA repair, which is crucial for cancer prevention. This is especially apparent in individuals with germline defects in the FA pathway, who are dramatically more likely to develop HNSCCs with early onset and an aggressive disease course characterized by EMT-like phenotypes [17,18,19,24]. Heightened susceptibility is attributable, at least in part, to the critical role of the FA pathway in maintaining genomic integrity through DNA repair and in preventing the accumulation of the oncogenic mutations that drive cancer progression [12,52]. However, emerging evidence highlights broader roles for the FA pathway in maintaining cellular homeostasis beyond its canonical function in ICL repair [53]. These include genome-stabilizing activities such as replication fork stabilization and suppression of aberrant origin firing, as well as cellular quality control mechanisms such as selective autophagy of damaged organelles and viral components [54,55]. Additionally, FA proteins like FANCD2 and FANCP are involved in transcriptional regulation and metabolism [56,57,58]. For example, FANCD2 represses the transcription of Peroxisome Proliferator-Activated Receptor-γ (PPARγ), a regulator of lipid metabolism, influencing the expression of genes related to lipid storage, glucose metabolism, and inflammation [59]. This highlights a multifaceted role of the FA pathway in maintaining cellular and metabolic balance.

In this study, we uncover an additional non-canonical role of the FA pathway in limiting protein synthesis by suppressing amino acid production and mTOR-driven translation. This is supported by our metabolomics analysis, which indicated that disruption of the FA pathway leads to elevated levels of several amino acids known to activate mTOR signaling (Figure 1). Notably, this included leucine, one of the most potent mTOR activators, as well as less potent activators such as glutamine, valine, threonine, alanine, and isoleucine [60,61,62]. These likely contribute to the heightened mTOR activity observed in FA− deficient HNSCC cells, thereby stimulating downstream effectors 4E-BP1 and S6 kinases (Figure 2). The resulting cascade promoted elevated translation and protein synthesis (Figure 2 and Figure 3), processes that are frequently hijacked in cancer to fuel unchecked proliferation and cancer progression. By establishing a mechanistic link between FA loss and amino acid-driven mTOR activation, our findings uncover a new metabolic vulnerability in FA− deficient HNSCCs.

Given that aberrant mTOR signaling is a well-established driver of tumor growth, EMT, metastasis, and resistance to cell death [63,64,65,66], targeting this pathway represents a compelling therapeutic strategy for preventing the progression of FA− deficient cancers. Rapamycin, a widely studied mTOR inhibitor, exhibits context-dependent effects influenced by tissue type, disease context, and treatment duration and dose [50,67]. In FA− deficient HNSCC cells, we observed a dose-dependent sensitization to rapamycin (Figure 5), likely influenced by the differential sensitivity of downstream targets—S6 is dephosphorylated at nanomolar concentrations, whereas 4E-BP1 is dephosphorylated at micromolar concentrations [68,69], which may explain more pronounced effects at higher doses. Although rapamycin primarily targets mTORC1, prolonged exposure (24–48 h) can also impair mTORC2 function by sequestering newly synthesized mTOR proteins [70,71,72], potentially contributing to the sensitization observed after 48 h of treatment.

Importantly, our findings demonstrate that rapamycin sensitivity in FA− deficient HNSCC cells is dependent on nutrient deprivation, including the depletion of amino acids, suggesting a heightened sensitivity to translational inhibition when nutrient availability is limited. This observation is particularly relevant given that tumor cells frequently encounter nutrient stress in vivo yet must maintain elevated biosynthetic activity to support proliferation, invasion, and metastasis [73,74]. Such metabolic pressure may amplify the therapeutic efficacy of rapamycin in the context of FA deficiency.

Since joint nutrient deprivation and mTOR inhibition appear to synergistically impair cellular growth, we propose that chemical or dietary amino acid/nutrient restriction could be explored as a potential adjuvant strategy to enhance the therapeutic efficacy of rapamycin in FA− deficient HNSCCs, an approach supported by the promise of dietary interventions in other cancer contexts [75,76]. Numerous studies on head and neck cancer have highlighted the influence of diet and nutrition on clinical outcomes, not only as prognostic indicators but also as modulators of treatment toxicity and efficacy [77,78,79,80]. This growing body of evidence supports the notion that targeted amino acid/nutrient restriction may represent a promising avenue for refining nutritional biomarkers and optimizing therapeutic strategies in this patient population [80].

Rapamycin has also been implicated in the induction of DNA damage through inhibition of double-strand break repair and promotion of apoptosis in certain contexts [69,81,82]. Since patients with FA are born with inherent defects in DNA repair, treatment with rapamycin warrants careful consideration. However, we did not observe a corresponding increase in total protein levels in FA− deficient normal cells (Figure 1C), suggesting that FA loss in non-malignant contexts may not activate mTOR signaling to the same extent—potentially sparing normal tissues from the effects of rapamycin. This distinction supports the therapeutic promise of rapamycin in patients with FA, who urgently need cancer treatments with minimal toxicity to healthy cells. Preclinical studies will be necessary to definitively rule out possible side effects of rapamycin on normal mucosal and skin cells for safety considerations in the FA disease context. These concerns may be less significant in the general population, where FA mutations are typically confined to the tumor. This distinction enhances the feasibility of rapamycin as a therapeutic option, especially given its well-characterized and extensively studied clinical side effect profile.

Several lines of evidence suggest broad implications across diverse cancer types. First, patients with FA are predisposed not only to SCCs but also to hematologic malignancies such as acute myeloid leukemia (AML) [15,16]. In FA− deficient bone marrow, fetal stem cell exhaustion and leukemogenesis have been linked to elevated protein synthesis, underscoring the potential for mTOR sensitization in FA− deficient leukemia [83]. Second, somatic mutations in FA genes are frequently observed in SCCs, as well as other malignancies such as bladder, pancreas, and lung cancers [84,85]. Third, key FA genes, including BRCA1 (FANCS) and BRCA2 (FANCD1), are well-established drivers of breast cancer [85,86]. Further investigation beyond squamous cell cancers will be required to test whether FA pathway loss leads to mTOR addiction under limited amino acid/nutrient supplies in other tumor types. Screens for FA loss-of-function mutations may then be more broadly applied to identify the subset of rapamycin-sensitive tumors.

**Figure 6:**
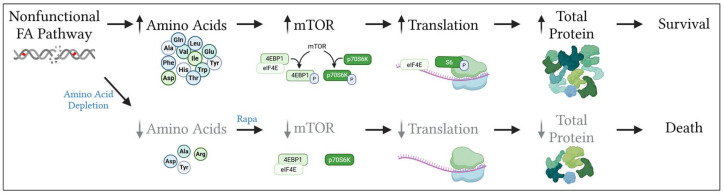
Working model: FA pathway deficiency induces amino acid production and mTOR-mediated translation. A dysfunctional FA pathway, associated with ICLs, leads to elevated intracellular amino acid levels that activate mTOR signaling, indicated by increased phosphorylation of 4E-BP1 and S6. This activation enhances translation to increase total protein content. Treatment with rapamycin decreases mTOR activation, suppresses translation, and reduces total protein levels. Under nutrient-deprived conditions, including amino acid limitations, rapamycin sensitizes FA− deficient HNSCC cells to growth inhibition. Combining amino acid deprivation with rapamycin treatment may represent a new therapeutic strategy for FA− HNSCCs. This figure was created with BioRender.com.

Given the therapeutic potential of mTOR inhibition in FA− deficient cancers, understanding the mechanistic basis of this sensitivity is of both biological and clinical significance. As depicted in the working model in Figure 6, we posit that elevated amino acid levels following FA pathway loss promote mTOR-dependent survival. However, the underlying mechanism remains unknown. Increased amino acid levels may result from the loss of classic FA pathway functions in repairing DNA ICLs and stabilizing replication forks, resulting in genome instability. Downstream DNA damage responses (DDRs) triggered by chronic genotoxic stress in FA− deficient HNSCC cells [87] may be involved in that case. For instance, the DDR effector p53 has been reported to be hyperactive in FA− deficient bone marrow [88,89], and in an unrelated study, unrestrained p53 activity was shown to drive amino acid biosynthesis, transport, and salvage pathways [90]. Alternatively, physical interactions between mTORC1 and FANCD2 have been reported in the context of genome instability and replication stress [81,91,92,93]. Defective mTORC1/FANCD2 complex formation or the loss of non-canonical FA pathway functions [53,56] may be involved in amino acid metabolism via yet unknown mechanisms.

## 5. Limitations

First, this study is limited by the use of cultured monolayer cells, which do not fully capture the 3D tumor structure and complexity of the tumor microenvironment nor in vivo conditions. Second, the observed rapamycin sensitization in FA− deficient HNSCC cells was performed in media deprived of all amino acids and multiple nutrients (Figure 5C); the importance of specific amino acids or nutrients was not further examined. Third, while our data demonstrated that FA− deficient normal squamous cells are not susceptible to mTOR inhibition (Figure 1C), this conclusion is constrained by the limited sensitivity of the BCA assay. More precise analyses will be essential to validate the unique metabolic dependencies of FA− deficient cancers and, by extension, the lack of rapamycin toxicity in the normal FA− tissue of persons with FA.

## 6. Conclusions

Our study reveals a novel function of the FA pathway in suppressing mTOR signaling and protein translation, with important implications for cancer biology and therapeutic intervention. We show that loss of FA function leads to amino acid accumulation, mTOR hyperactivation, and increased protein synthesis, creating metabolic vulnerabilities to translational inhibition in FA− deficient cells. These findings support a novel mechanism, whereby the FA pathway may suppress tumor growth and progression, while also uncovering a previously underexplored metabolism-based treatment. Specifically, our results support the combination of mTOR inhibitors with nutrient restriction as a targeted strategy for treating FA− deficient HNSCCs. Future in vivo studies will be essential to validate these findings and to further define the broader impact of FA–mTOR interactions on tumor development, progression, and therapeutic response. Such models will enable a more comprehensive evaluation of the efficacy and safety of mTOR-targeted therapies like rapamycin, both in the general population and in individuals with FA.

## Data Availability

The original contributions presented in this study are included in the article/Supplementary Material. Further inquiries can be directed to the corresponding author.

## Supplementary Materials

The following supporting information can be downloaded at: https://www.mdpi.com/article/10.3390/cancers17152583/s1, Figure S1: Western blots for Figure 2B with associated densitometry values; Figure S2: Western blots for Figure 2C with associated densitometry values; Figure S3: Western blots for Figure 3A with associated densitometry values; Figure S4: FA pathway deficiency inhibits FA− HNSCC cell growth under nutrientdepleted conditions.

## Abbreviations

2D	Two-dimensional
3D	Three-dimensional
4E-BP1	4E binding protein 1
AML	Acute myeloid leukemia
ANOVA	Analysis of variance
BCA	Bicinchoninic acid
BSA	Bovine serum albumin
CC3	Cleaved caspase-3
D2sh	Short-hairpin RNA-treated cells targeted for FANCD2
DDR	DNA damage response
EdU	5-ethynyl-2′-deoxyuridine; a nucleotide analog used to quantify cellular proliferation
eIF4/2	Eukaryotic initiation factor-4 or -2
EMT	Epithelial-to-mesenchymal transition
ESI	Electrospray ionization
FA	Fanconi anemia; a genetic disease and DNA repair pathway
FA+	Fanconi anemia pathway-proficient; indicates cells have a functional FA pathway
FA−	Fanconi anemia pathway-deficient; indicates cells have a nonfunctional FA pathway
GSD	Global spectra deconvolution
HMDB	Human Metabolome Database
HNSCC	Head and neck squamous cell carcinoma
HPG	L-homopropargylglycine; a methionine analog used to quantify cellular translation
HR	Homologous recombination
HSQC	Heteronuclear single quantum correlation
ICL	DNA interstrand crosslinks
ID2 kDa	The FANCI/FANCD2 intermediate complex in the Fanconi anemia pathway kilodalton
mTOR	Mechanistic target of rapamycin
MS	Mass spectrometry
NIKS	Normal immortalized keratinocytes
NMR	Nuclear magnetic resonance
NOKS	Normal oral mucosal keratinocytes
NTsh	Non-targeting short-hairpin RNA-treated cells
PBS	Phosphate-buffered saline
PFA	Paraformaldehyde
PPARγ	Peroxisome proliferator-activated receptor-γ
RIPA	Radioimmunoprecipitation assay
SCC	Squamous cell carcinoma
TBST	Tris-buffered saline with Tween 20
TSP	Trimethylsilyl propionic acid-d4 sodium salt
UHPLC	Ultra-high performance liquid chromatography
WGA	Wheat germ agglutinin
