# Supplementary material for: The Fanconi Anemia Pathway Inhibits mTOR Signaling and Prevents Accelerated Translation in Head and Neck Cancer Cells
Source: Cancers (Basel). 2025 Aug 6;17(15):2583. doi: 10.3390/cancers17152583 (PMC12346401; doi:10.3390/cancers17152583)
Supplement: Supplementary file 1 [file cancers-17-02583-s001.zip › cancers-3733603-supplementary.pdf]

Supplemental Figure 1. Western blots for Figure 2B with associated densitometry values.

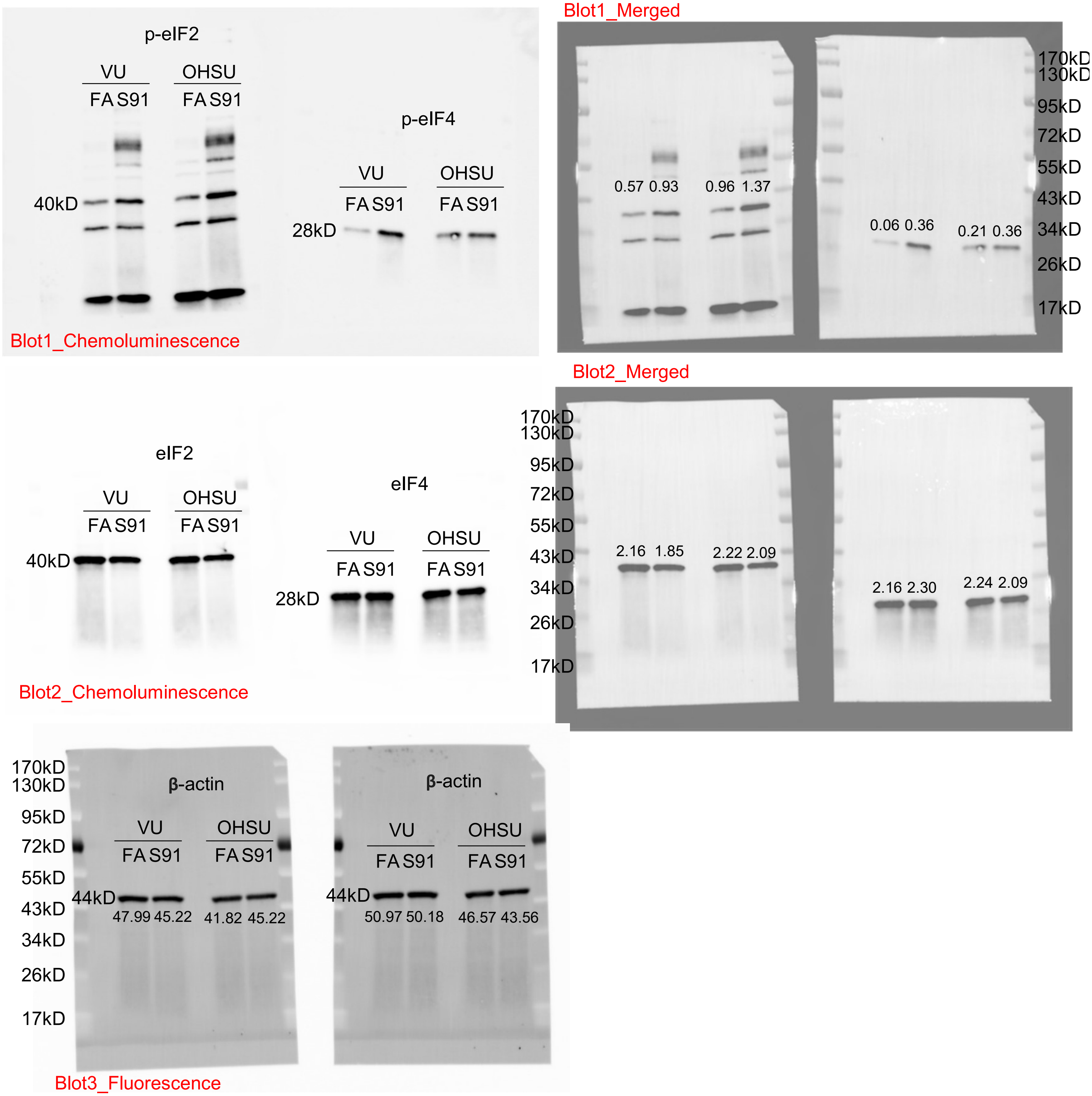

Supplemental Figure 1. Western blots for Figure 2B with associated densitometry values.

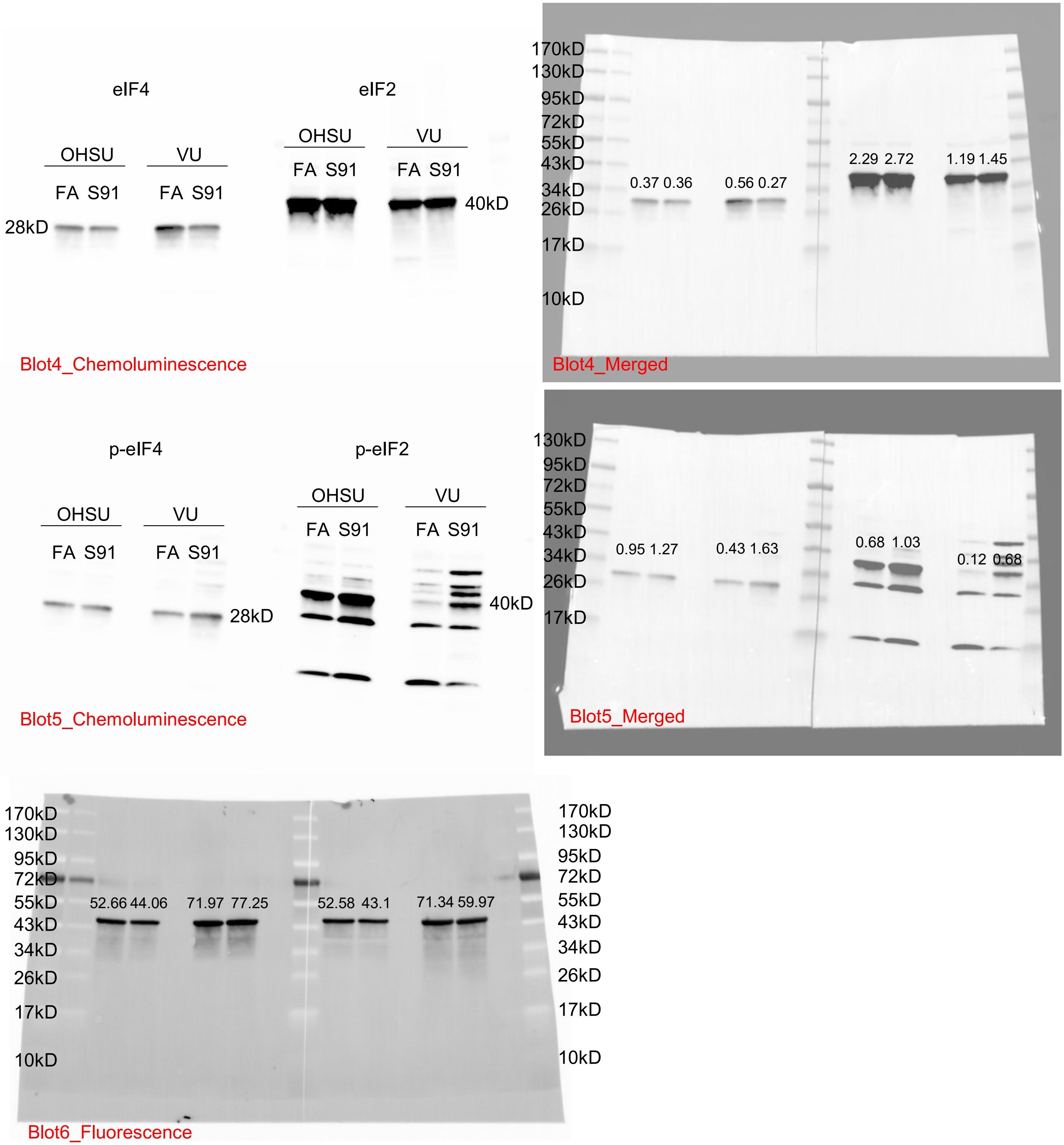

Supplemental Figure 1. Western blots for Figure 2B with associated densitometry values.

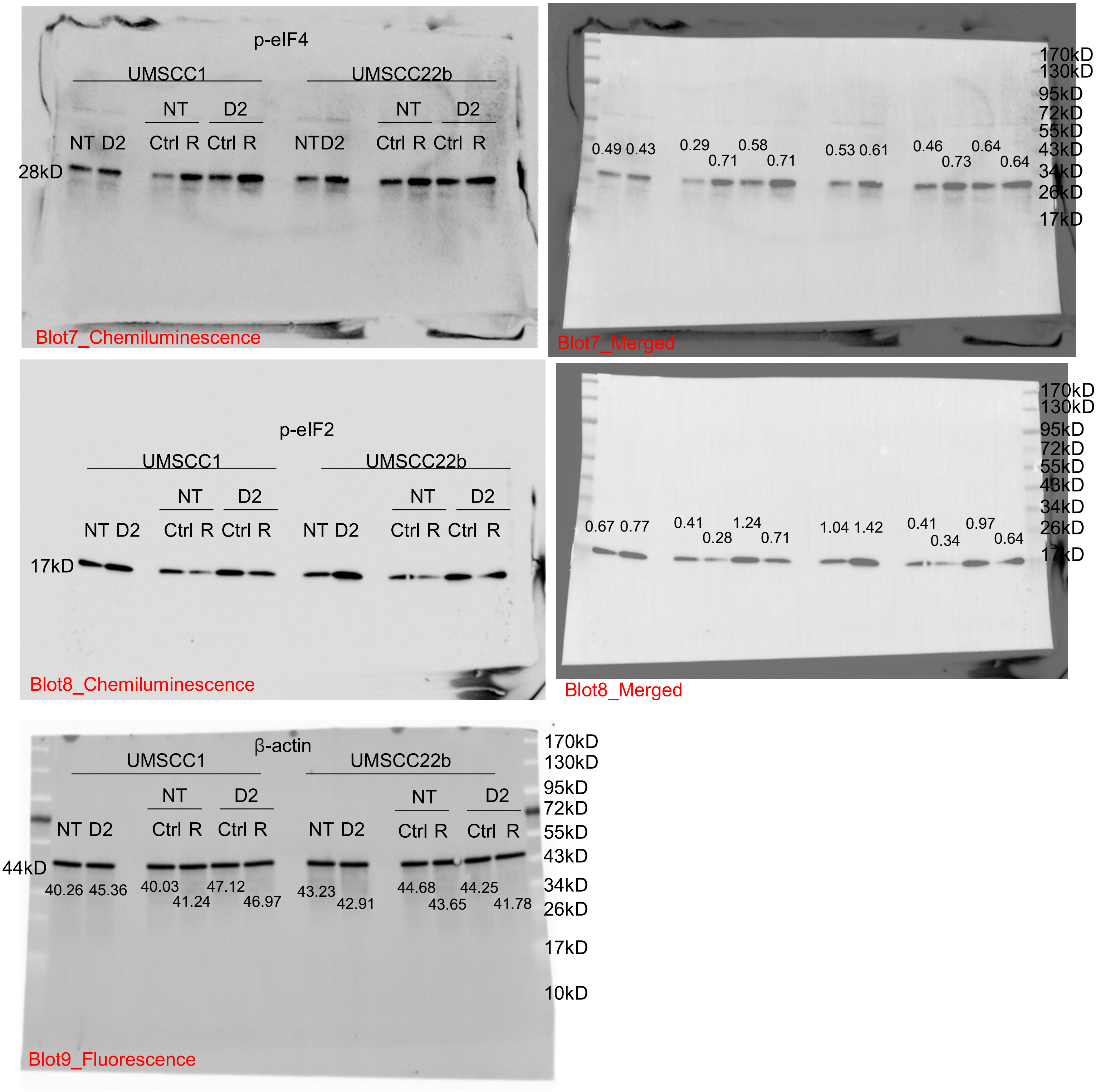

Supplemental Figure 1. Western blots for Figure 2B with associated densitometry values.

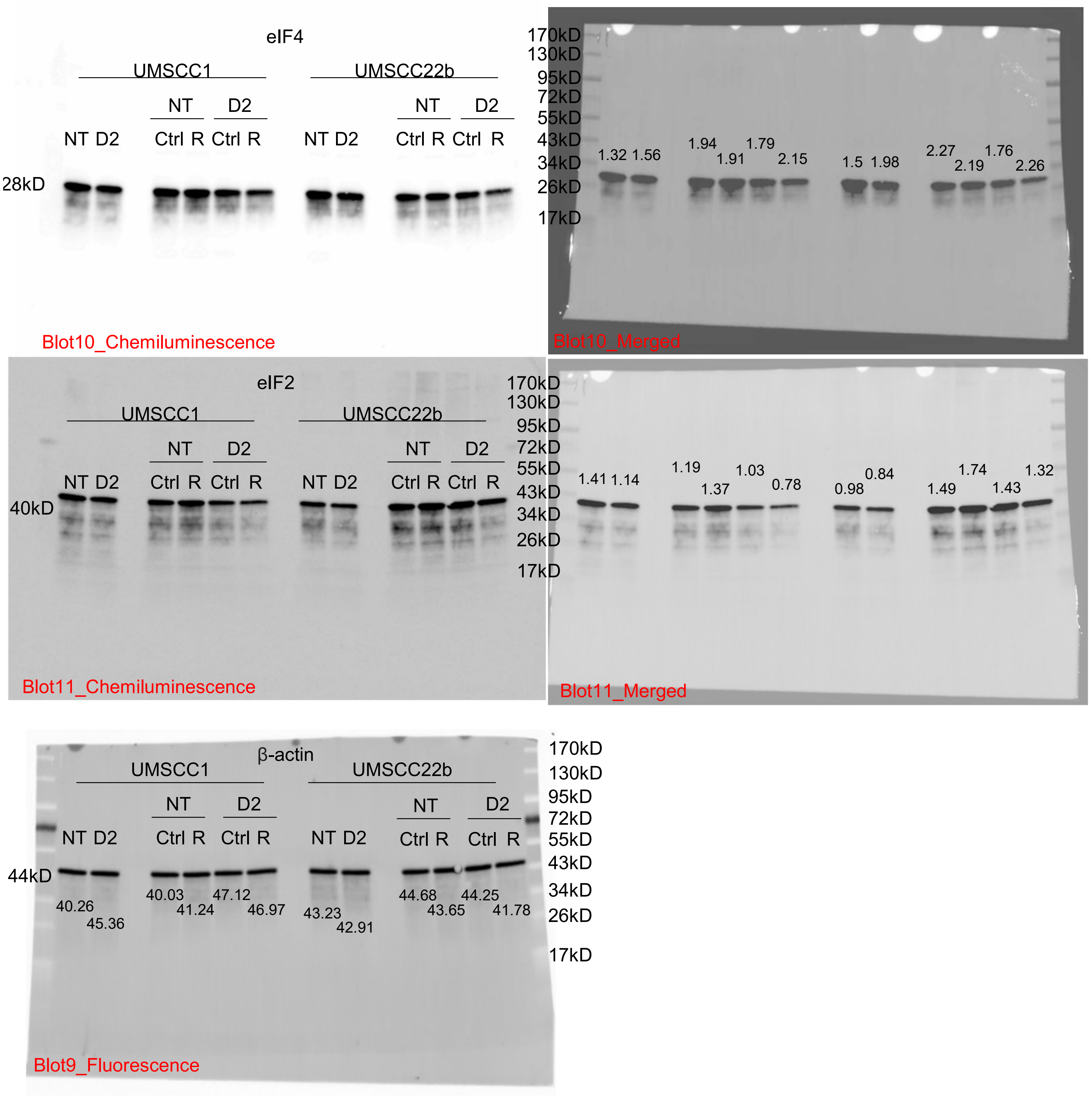

Supplemental Figure 1. Western blots for Figure 2B with associated densitometry values.

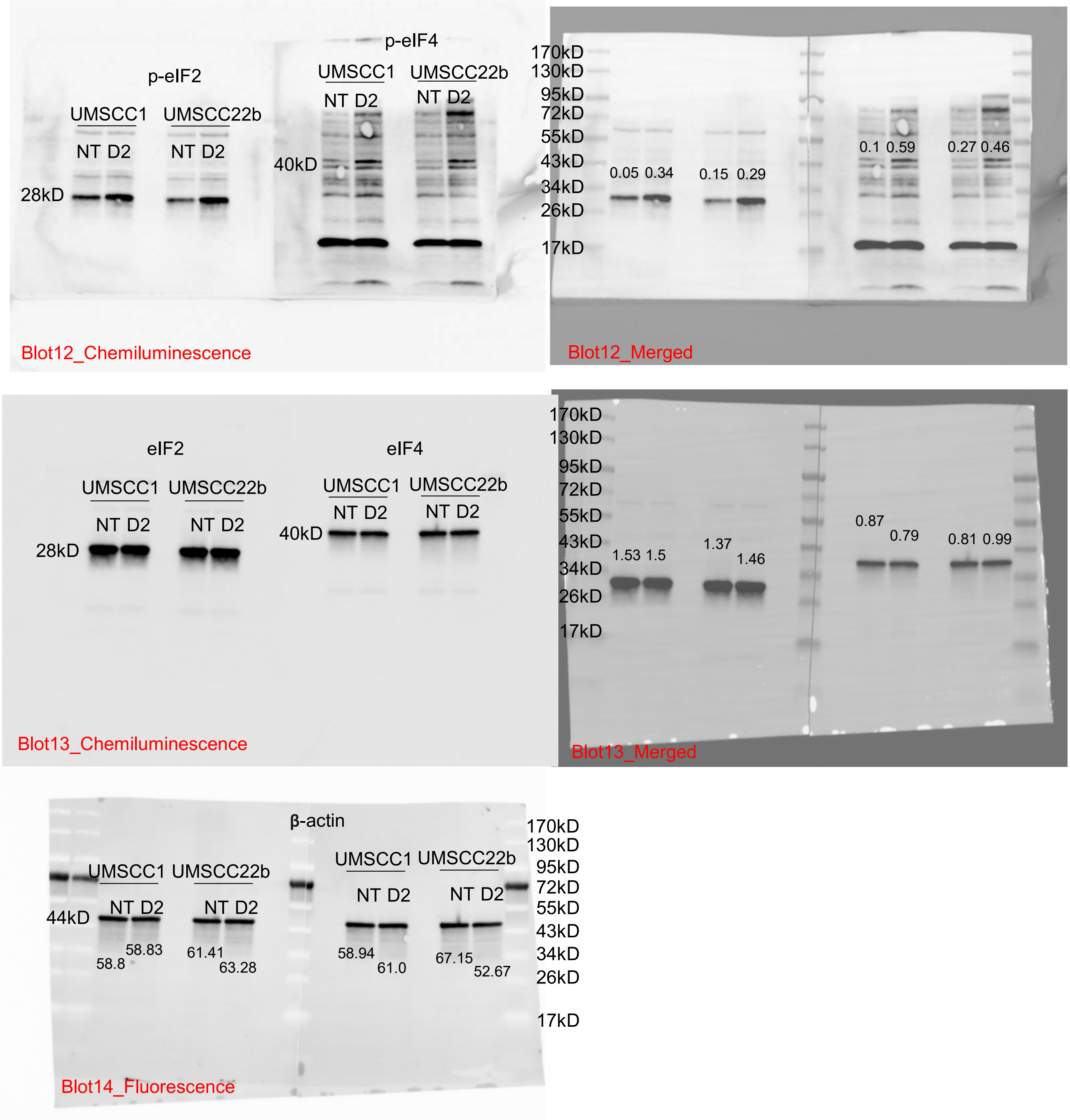

Supplemental Figure 2. Western blots for Figure 2C with associated densitometry values.

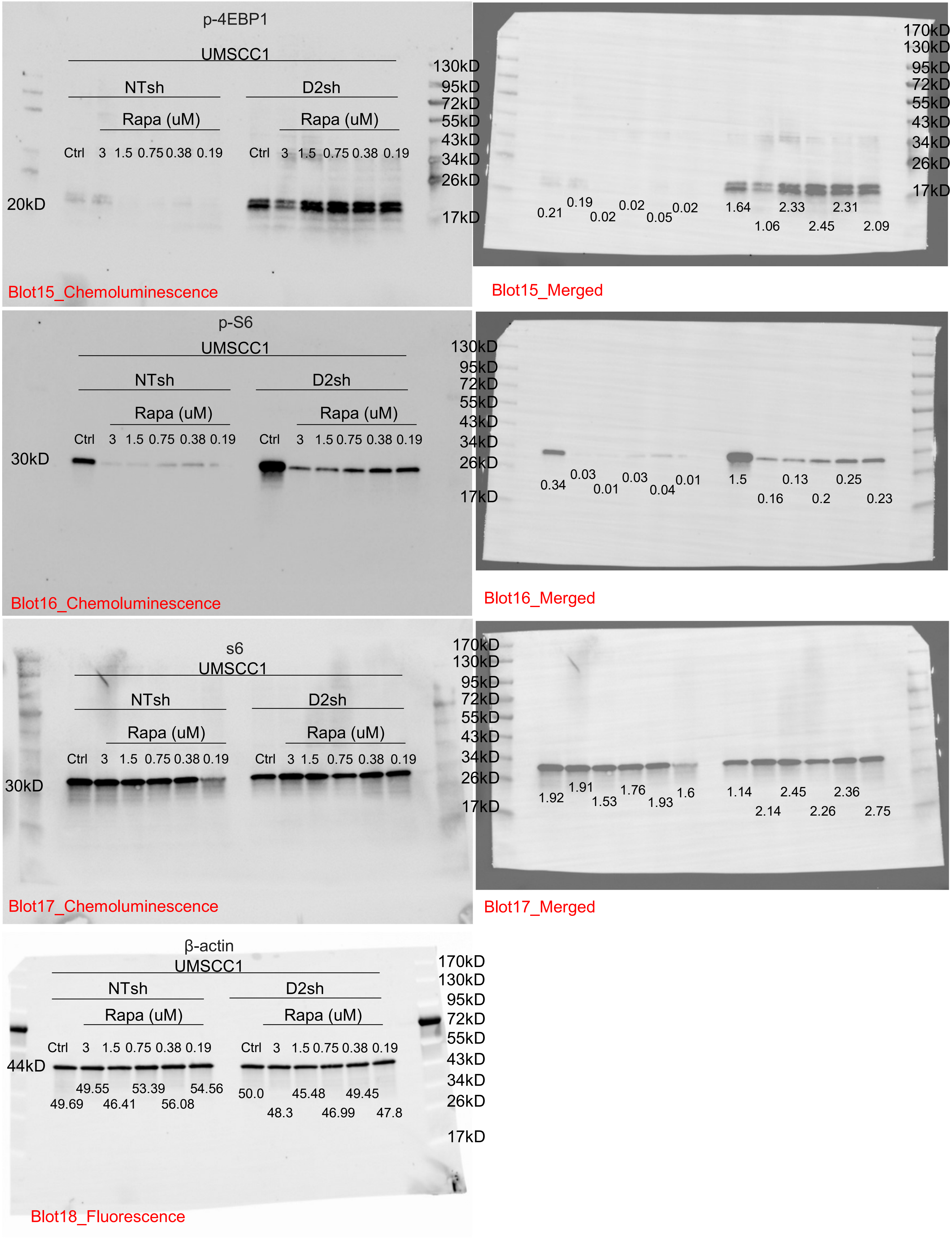

Supplemental Figure 2. Western blots for Figure 2C with associated densitometry values.

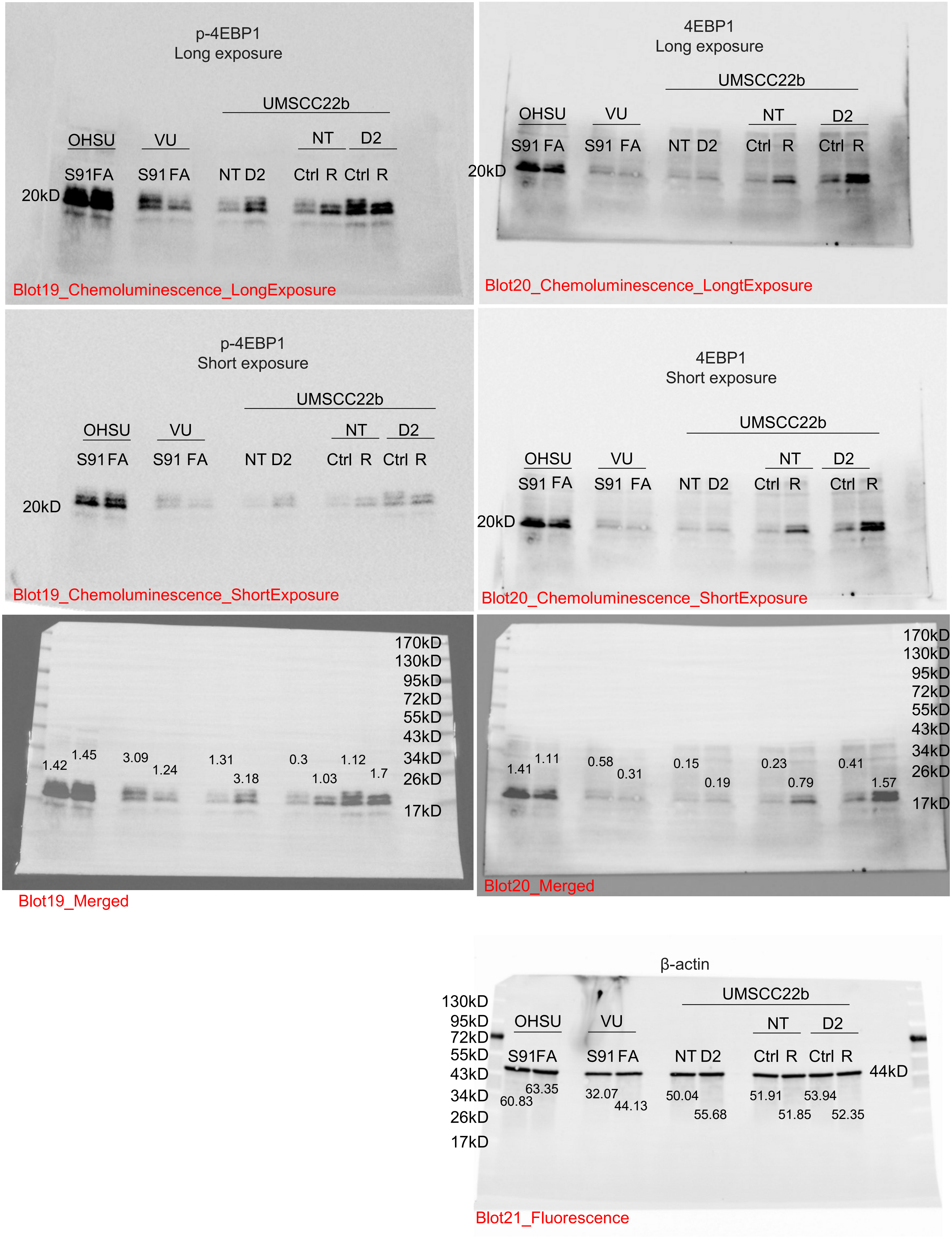

Supplemental Figure 2. Western blots for Figure 2C with associated densitometry values.

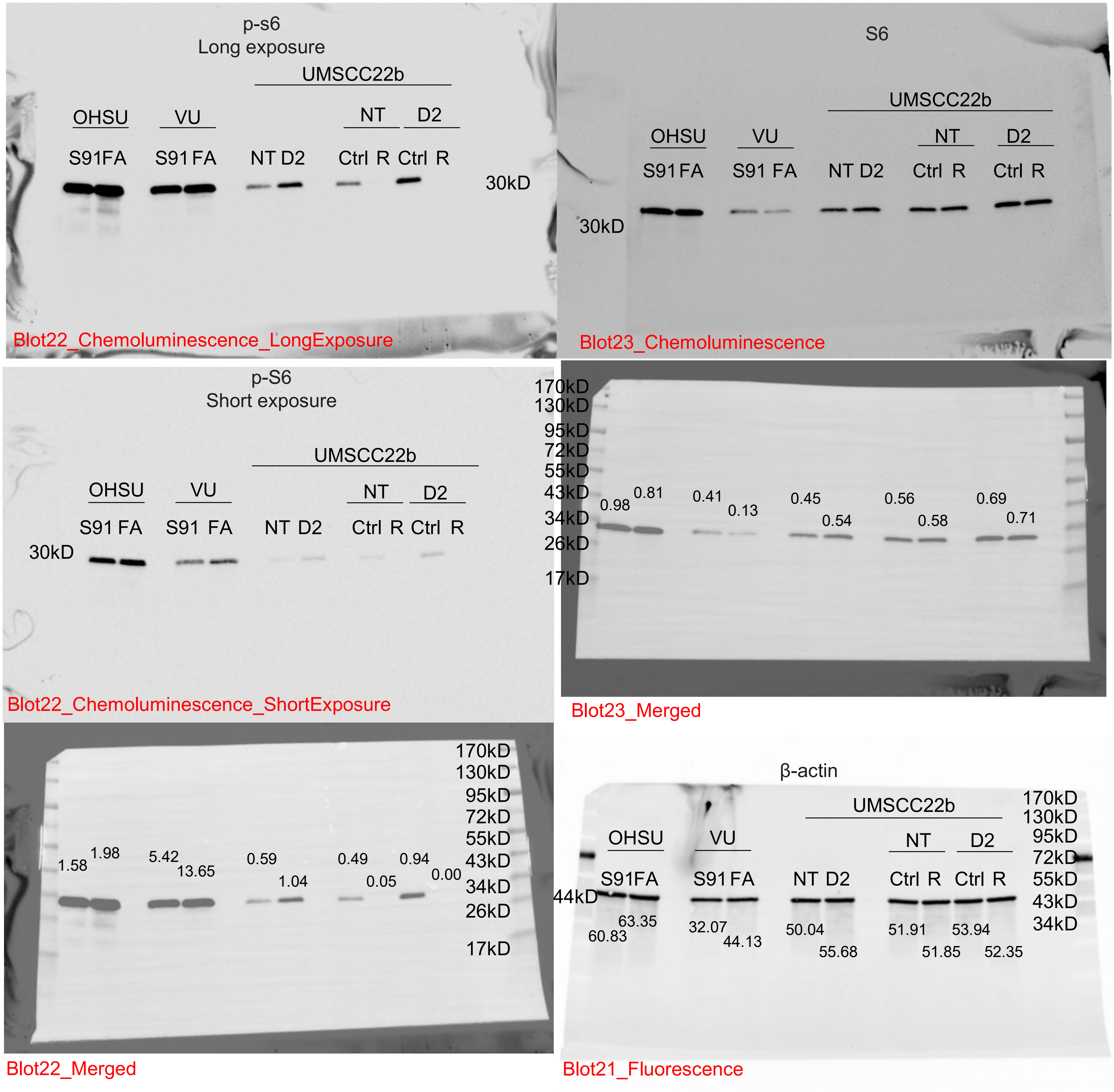

Supplemental Figure 2. Western blots for Figure 2C with associated densitometry values.

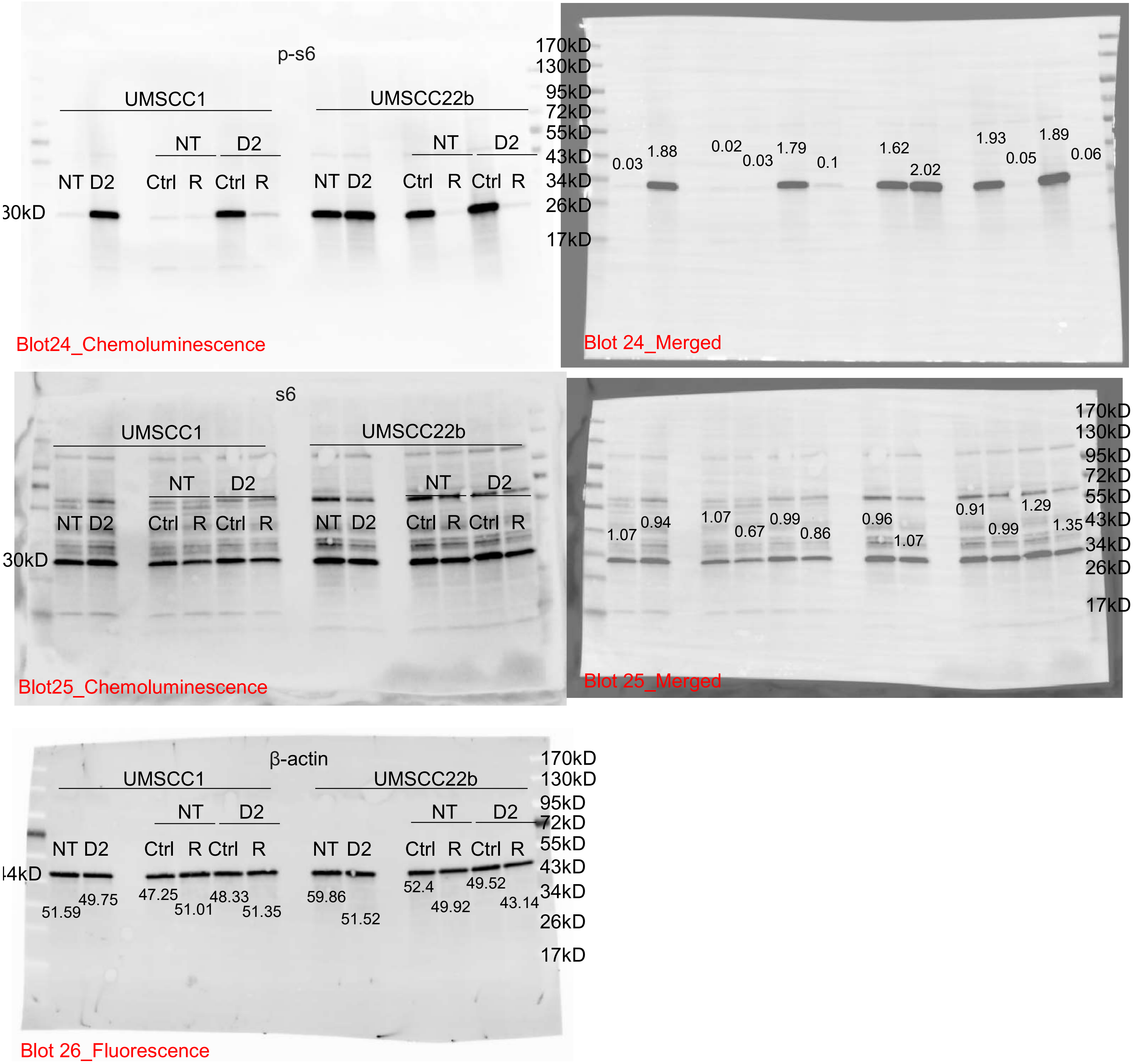

Supplemental Figure 2. Western blots for Figure 2C with associated densitometry values.

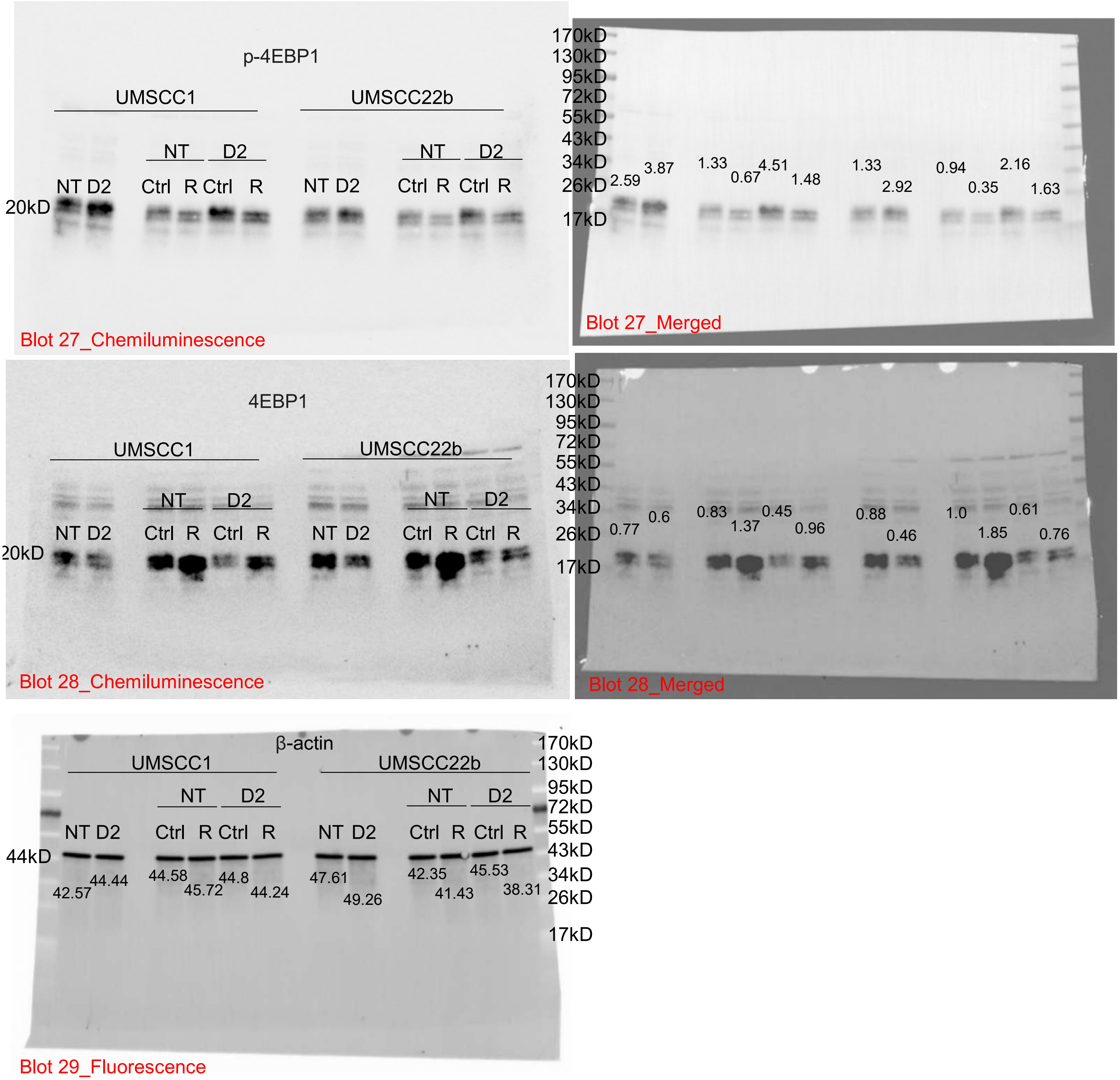

Supplemental Figure 2. Western blots for Figure 2C with associated densitometry values.

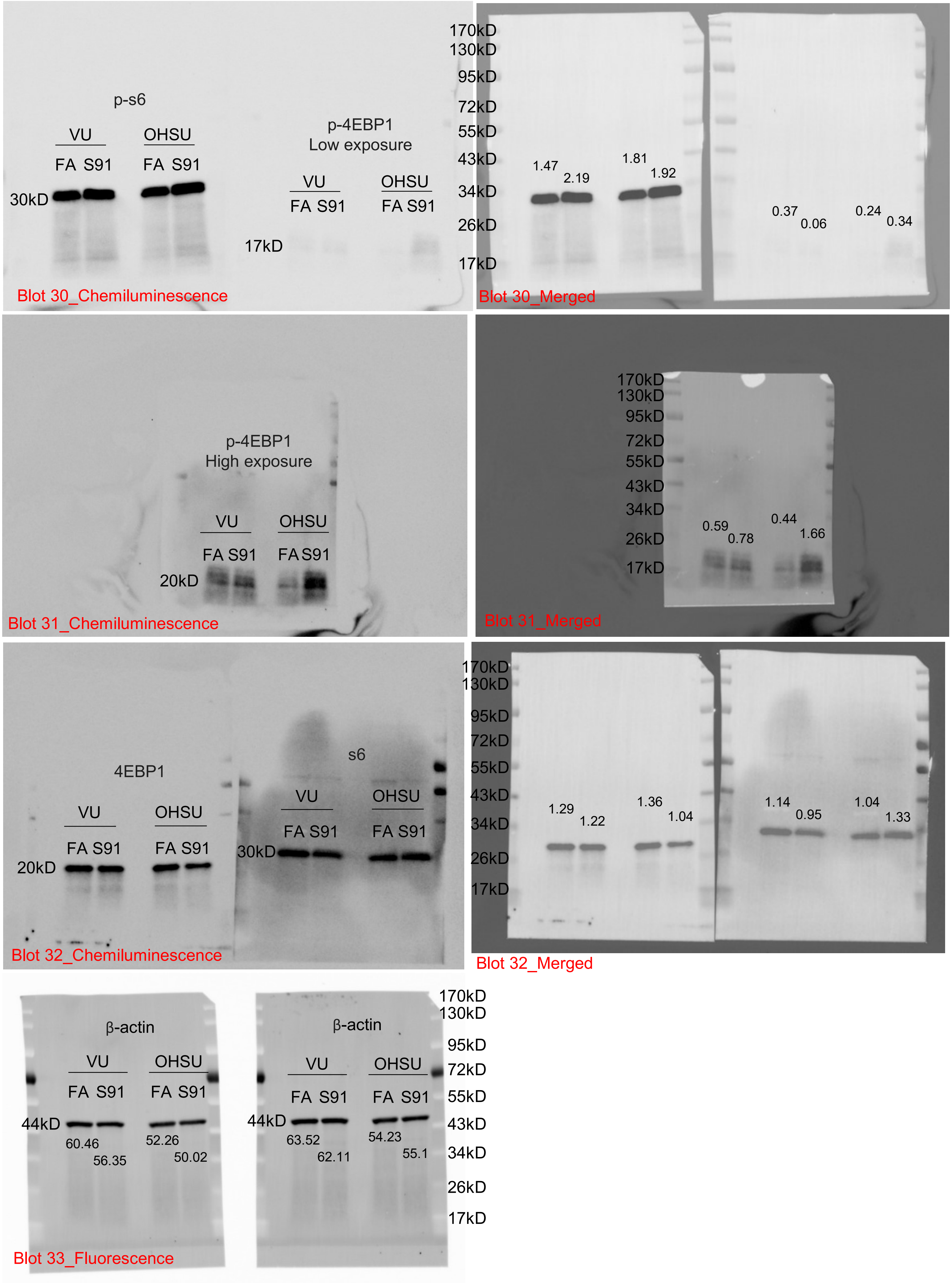

Supplemental Figure 3. Western blots for Figure 3A with associated densitometry values.

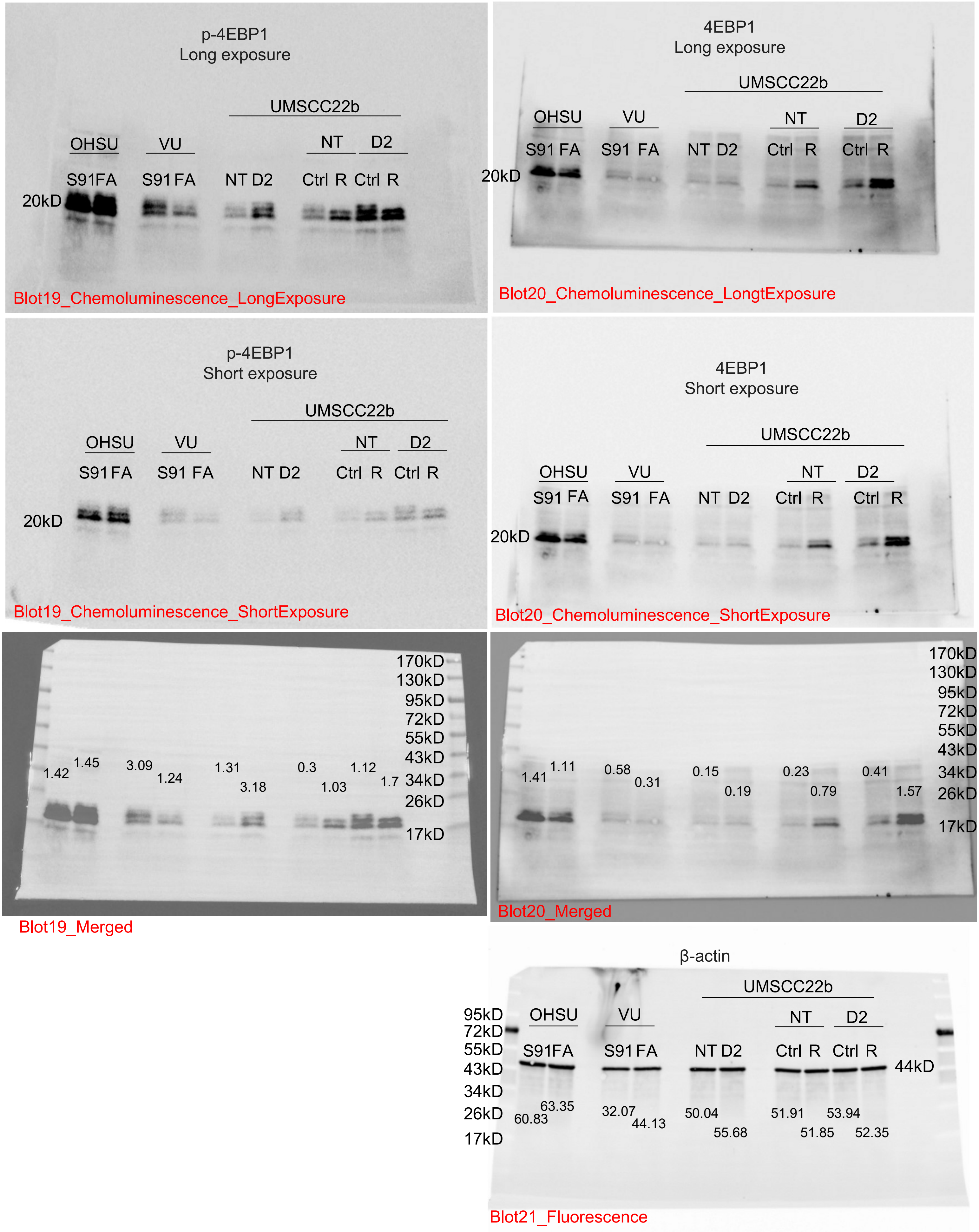

Supplemental Figure 3. Western blots for Figure 3A with associated densitometry values.

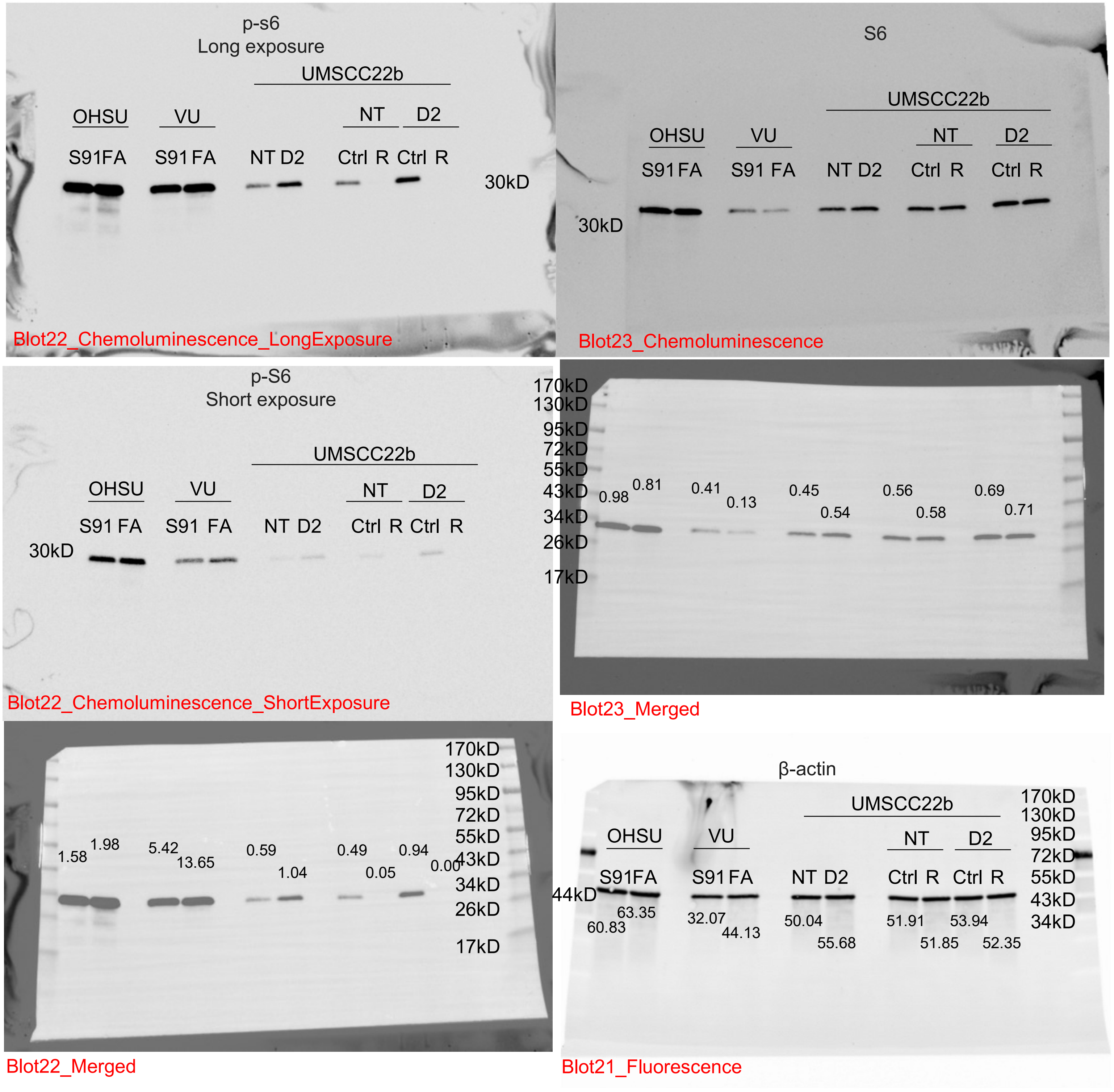

Supplemental Figure 3. Western blots for Figure 3A with associated densitometry values.

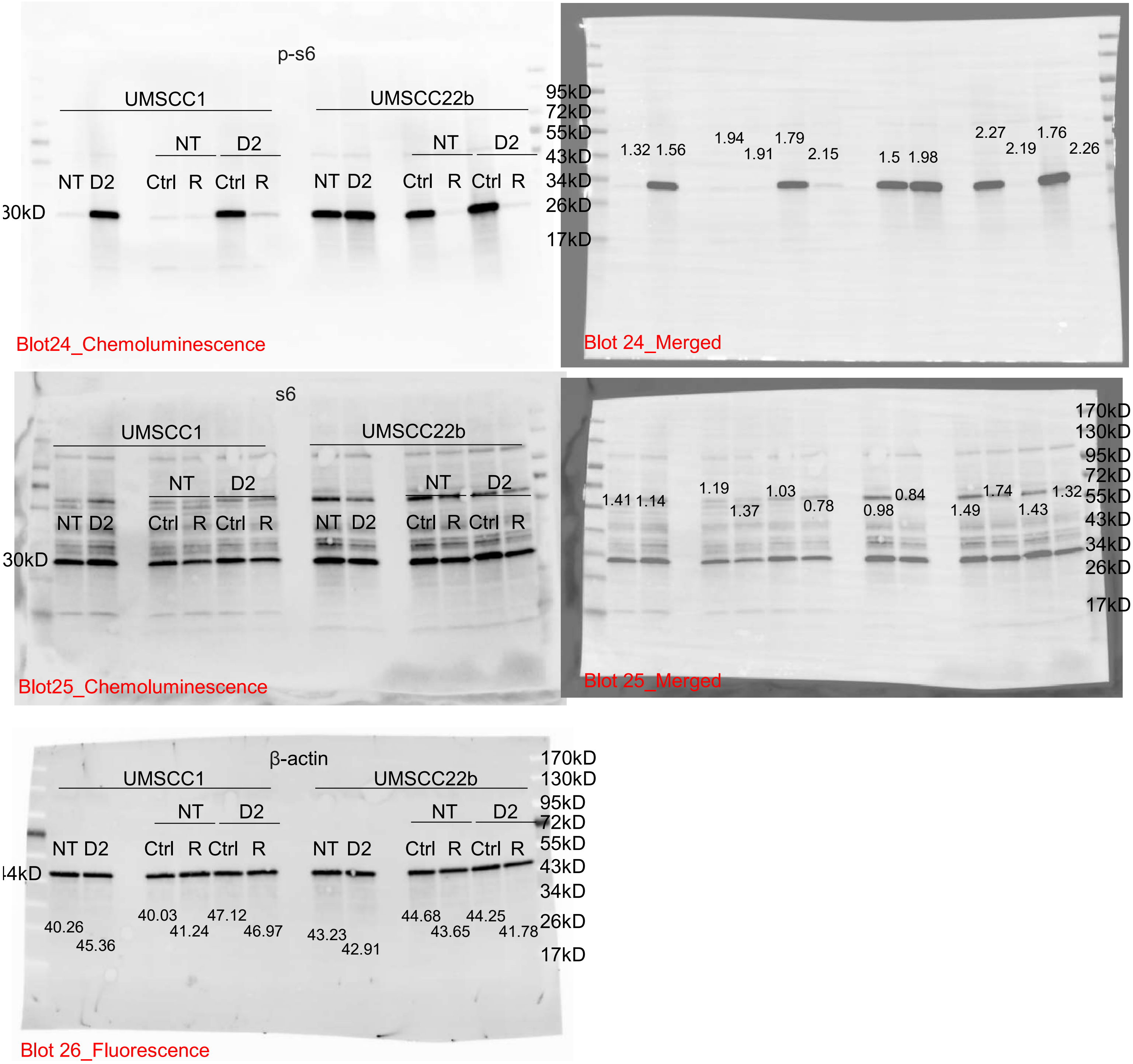

Supplemental Figure 3. Western blots for Figure 3A with associated densitometry values.

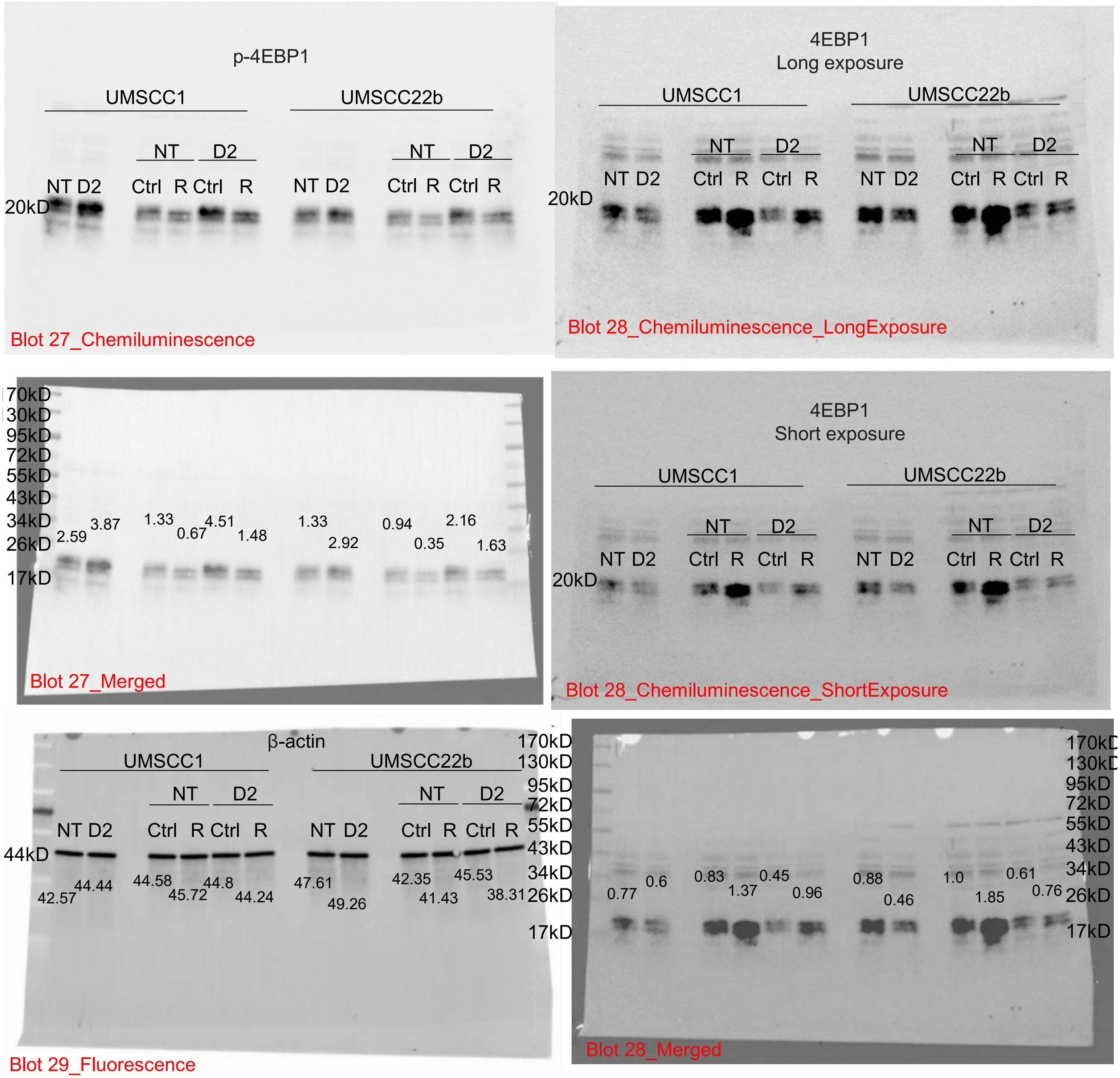

Supplemental Figure 4. FA pathway deficiency inhibits FA- HNSCC cell growth under nutrient-depleted conditions.

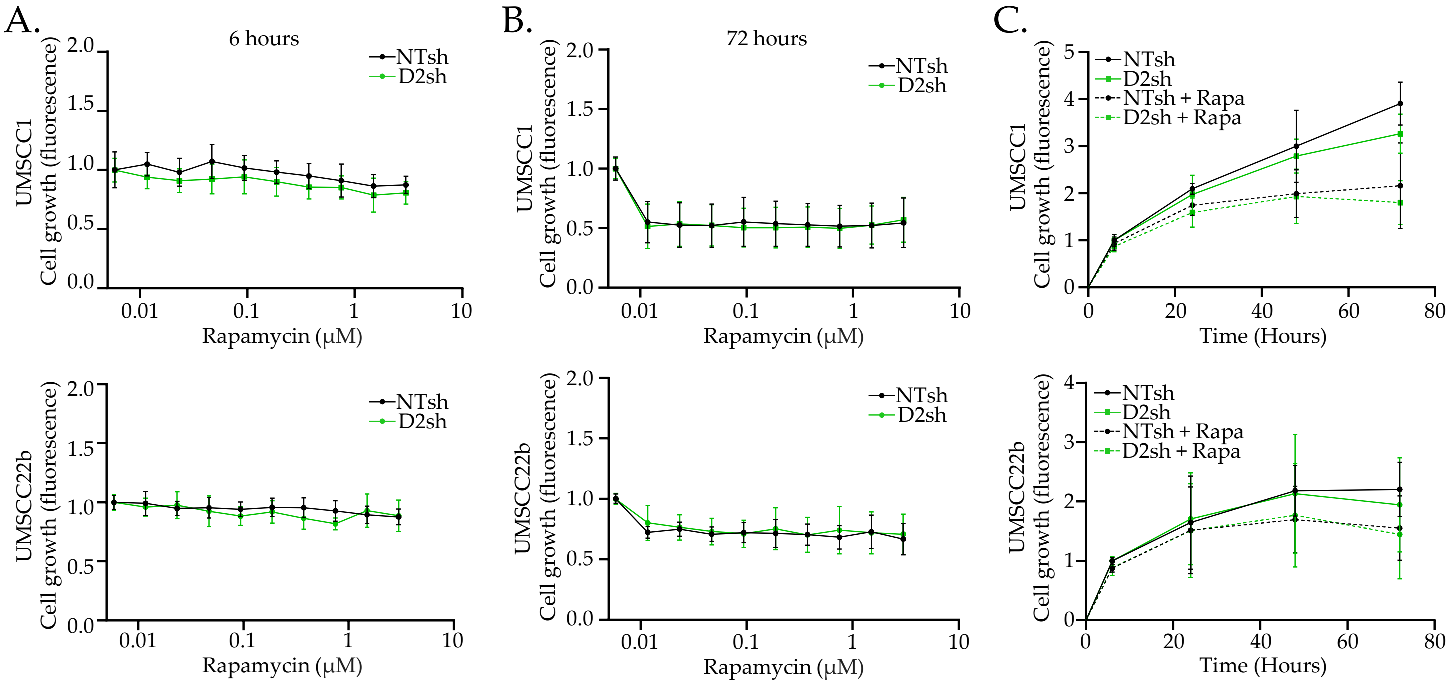

Figure Supplementary 4. Rapamycin sensitivity is independent of FA status under nutrient-rich conditions. Cell viability was assessed using the CellTiter-Fluor assay following (**A**) 6-hour and (**B**) 72-hour treatments with rapamycin or PBS and (**C**) over time with 3 μM rapamycin treatment in nutrient-rich media (DMEM). Data represent mean ± SD (n = 3). Differences in growth curves were assessed using two-way ANOVA comparing NTsh and D2sh growth at each concentration of rapamycin. Absence of a symbol indicates no significant difference.
